# A comparison of seven random‐effects models for meta‐analyses that estimate the summary odds ratio

**DOI:** 10.1002/sim.7588

**Published:** 2018-01-08

**Authors:** Dan Jackson, Martin Law, Theo Stijnen, Wolfgang Viechtbauer, Ian R. White

**Affiliations:** ^1^ MRC Biostatistics Unit, Institute of Public Health University of Cambridge Cambridge UK; ^2^ Medical Statistics and Bioinformatics Leiden University Medical Center Leiden Netherlands; ^3^ Psychiatry and Psychology Maastricht University Maastricht Netherlands; ^4^ MRC Clinical Trials Unit Institute of Clinical Trials and Methodology University College London London UK

**Keywords:** binomial distribution, exact within‐study distributions, random‐effects models, statistical computing

## Abstract

Comparative trials that report binary outcome data are commonly pooled in systematic reviews and meta‐analyses. This type of data can be presented as a series of 2‐by‐2 tables. The pooled odds ratio is often presented as the outcome of primary interest in the resulting meta‐analysis. We examine the use of 7 models for random‐effects meta‐analyses that have been proposed for this purpose. The first of these models is the conventional one that uses normal within‐study approximations and a 2‐stage approach. The other models are generalised linear mixed models that perform the analysis in 1 stage and have the potential to provide more accurate inference. We explore the implications of using these 7 models in the context of a Cochrane Review, and we also perform a simulation study. We conclude that generalised linear mixed models can result in better statistical inference than the conventional 2‐stage approach but also that this type of model presents issues and difficulties. These challenges include more demanding numerical methods and determining the best way to model study specific baseline risks. One possible approach for analysts is to specify a primary model prior to performing the systematic review but also to present the results using other models in a sensitivity analysis. Only one of the models that we investigate is found to perform poorly so that any of the other models could be considered for either the primary or the sensitivity analysis.

## 1 INTRODUCTION

Meta‐analysis is widely used in a variety of application areas, including medicine, and now requires very little introduction. Data from multiple comparative trials involving binary outcomes are very often pooled in meta‐analyses. For example, Davey et al^1^ found 14 886 meta‐analyses with binary outcomes in the January 2008 issue of the Cochrane Database of Systematic Reviews. The observation that so many meta‐analyses of this type can be found within the Cochrane database provides clear evidence that the situation we examine here is extremely common. We assume that all trials compare the same pair of conditions or treatments and that there is an event of interest that gives rise to a binary outcome. Data of this type can be set out as a series of 2‐by‐2 tables. Our aim is to make inferences about which group is more likely to experience this event. A typical example is data from several randomised controlled trials, each with a treatment and a control group, and where the event of interest is death.

We will use random‐effects models to describe this type of data, which means that we can incorporate between‐study heterogeneity into our modelling. Heterogeneity in the treatment effects is likely to occur in practice due to the inclusion of patients from different patient populations, and variation in other circumstances that might influence outcomes, across the studies. If the strong assumption that the studies estimate the same true effect is made, then a common‐effect model may be used. We suggest that random‐effects models are in general to be preferred to common‐effect models because their assumptions are more plausible. However, determining which type of random‐effects model should be used in application is not straightforward and is the main subject of this paper. We will make some recommendations concerning this in the discussion.

A variety of outcome measures are available for this type of data.(2, 3, 4, 5, 6) These measures include the relative risk, the risk difference and the recently proposed arcsine difference. Here, we examine random‐effects models for the meta‐analysis of comparative binary data where the outcome measure is the odds ratio. The odds ratio is a very popular measure of treatment effect for meta‐analysis, and for statistical analysis more generally, because of its favourable statistical properties. The relative risk is another relative measure of treatment effect that, unlike the odds ratio, does not have the advantage of being invariant to the labelling of the event. This means that the relative risk of experiencing the event does not equal the relative risk of not experiencing the event. Another advantage of using odds ratios is that they are valid regardless of the type of sampling used, which is not the case for other comparative measures for binary data. However, determining the most appropriate statistical model to use when estimating the summary odds ratio is not a trivial decision and is the focus of this paper. The context of the application might also influence the analyst's model choice.

The most commonly used random‐effects methodology for estimating the summary odds ratio involves a 2‐stage approach. In the first stage, we calculate the study specific estimated log odds ratios and their (within‐study) standard errors. In the second stage, we assume that the conventional 2‐stage random‐effects model describes these estimates, so that we can pool them by taking a weighted average. The pooled estimate of the log odds ratio, and the corresponding confidence interval, can then be transformed onto the odds ratio scale. This procedure is justified on the basis that normal within‐study approximations are amenable to estimated log odds ratios, so that the conventional random‐effects model, and the corresponding usual inferential procedure,^7^ can be applied. However, the normal approximation made by the conventional random‐effects model can be poor when the studies are small or the event is rare. This can result in inaccurate inference.(8, 9)

More sophisticated models for random‐effects meta‐analysis,(10, 11, 12) that also result in a summary log odds ratio, and hence a summary odds ratio, have been proposed. These avoid using within‐study normal approximations and so in principle are preferable. However, as we will explain below, these methods introduce their own statistical issues. The aim of this paper is to compare the use of 7 different random‐effects models and assess their advantages and disadvantages. The first of these models is the conventional random‐effects modelling approach described above and the other models are different forms of generalised linear mixed models. It will be of particular interest to determine whether or not the more sophisticated models can improve upon the conventional model that is very widely used in application. If this is so, then the case for applied groups, such as Cochrane, updating their statistical practices is strong. If however the conventional model is found to be adequate then it may be that, rather than focussing on improving the statistical methods, such groups would better spend their energies on further improving other aspects of the systematic review process.

The rest of the paper is set out as follows. In Section 2, we show an illustrative dataset and we also introduce some mathematical notation. In Section 3, we describe the 7 statistical models that we examine in this paper. In Section 4, we discuss the issues and concerns that accompany the use of these 7 models. In Section 5, we explore the implications of using these models in the context of a Cochrane Review, and in Section 6 we perform a simulation study. We conclude in Section 7 with a short discussion.

## 2 AN ILLUSTRATIVE DATASET AND MATHEMATICAL NOTATION

We begin with an artificial example to show the type of data that will be analysed and the mathematical notation that will be used to describe the statistical models. In Table 1, we show an illustrative dataset that involves 4 studies. Each study involves both treatment groups so that there are 8 observations in total. Let i=1⋯k denote the study and j=0,1 denote the treatment group, where j=0,1 indicate the “control” and “treated” group, respectively. We denote the number of patients and events in the ith study and jth treatment as n_ij_ and e_ij_, respectively. We use covariates j, x_ij_, and z_ij_ in our models, where x_ij_ is an indicator for the control group and z_ij_=j−0.5. In the column headings of Table 1, as well as showing our mathematical notation, we also show the column names that will be used in our computing syntax. For example, in Table 1, we can see that j, the indicator for the treatment group, is referred to as “treat” in the computing syntax. These alternative ways of expressing important quantities mean that we can use conventional mathematical notation when presenting statistical models and also meaningful syntax when fitting them. The statistical package R^13^ will be used to perform all computing, where our R dataframes have the second set of column names shown in Table 1.

**Table 1:** An illustrative dataset. Two sets of column headings are shown. The first set shows the mathematical notation used to describe statistical models. The second set shows the column names of the corresponding R data frame

Study (i)	Treatment (j)	n_ij_	e_ij_	x_ij_	z_ij_
study	treat	n	event	control	treat12
1	0	377	113	1	−0.5
1	1	377	128	0	0.5
2	0	40	4	1	−0.5
2	1	41	6	0	0.5
3	0	100	20	1	−0.5
3	1	101	22	0	0.5
4	0	1010	201	1	−0.5
4	1	1001	241	0	0.5

We use π_ij_ to denote the probability of an event in the ith study and jth treatment group. For many models, we will describe binomial data directly, like that in Table 1, and so we will often assume that e_ij_∼Bin(n_ij_,π_ij_) with a suitable expression for π_ij_.

An alternative way of setting out the data in Table 1 is shown in Table 2. Here, A and B are the number of events, and nonevents, in the treated group, respectively. Similarly, C and D are these same quantities in the control group. Table 2 is just a very simple rearrangement of the data shown in Table 1. This is necessary because the methods implemented by the metafor package,^14^ that constitute some of the methods that we will investigate below, require the data to be set out in this way. Version 1.9‐9 of metafor was used throughout.

**Table 2:** Another way to set out the illustrative dataset. This is the data format required by the metafor package to fit some models

Study (i)	A	B	C	D
1	128	249	113	264
2	6	35	4	36
3	22	79	20	80
4	241	760	201	809

## 3 SEVEN STATISTICAL MODELS

Now that we have introduced our notation, we will introduce 7 statistical models. The first model is the conventional random‐effects model that uses normal within‐study approximations for the study specific estimated log odds ratios. The other models are generalised linear mixed models. We consider these particular alternative models because they have either been previously suggested in the literature (models 2, 6, and 7), implemented in the metafor package^14^ in R (models 4 and 5) or involve a particularly natural combination of modelling ideas from other models (model 3). We restrict our investigation to these alternative models because they are closely related to the conventional random‐effects model, where we assume that the study specific true odds ratios, θ_i_, are normally distributed. We suspect therefore that applied analysts, who wish to consider an alternative to the conventional model, are most likely to adopt one of these models. However, further models and methods are also available for analysts who are prepared to deviate further from the more usual methods for random‐effects meta‐analysis, for example, see Kuss^15^ for a wide range of other possibilities.

### 3.1 Model 1: the conventional random‐effects model

The conventional random‐effects model uses 2 stages. In the first stage, we estimate the study specific empirical log odds ratios, y_i_, and their within‐study variances, $s_{i}^{2}$. In the second stage, we assume the conventional random‐effects model(7, 16): $y_{i}|\theta_{i}∼N(\theta_{i},s_{i}^{2})$ and θ_i_∼N(θ,τ ^2^), so that marginally $y_{i}∼N(\theta ,s_{i}^{2}+\tau^{2})$. We treat the $s_{i}^{2}$ as fixed and known. We refer to the distributional assumption for y_i_|θ_i_ as the within‐study normality assumption; all models that follow below avoid using this approximation. Here, θ_i_ is the true underlying log odds ratio for the ith study, so that θ_i_=logit(π_i1_)−logit(π_i0_), and τ ^2^=Var(θ_i_) is the between‐study variance. The parameter θ is the summary log odds ratio. We exponentiate estimates and bounds of confidence intervals for θ to obtain inferences on the odds ratio scale.

#### 3.1.1 Fitting model 1

In the notation of Table 2, the estimated log odds ratios are given by $y_{i}=log((A_{i}/B_{i})/(C_{i}/D_{i}))$. We also calculate the corresponding within‐study variances in the first stage; for this, we use the standard formula $s_{i}^{2}=1/A_{i}+1/B_{i}+1/C_{i}+1/D_{i}$. We will use the metafor escalc command (with its defaults) for these purposes throughout. This means that halves are added to all counts in 2‐by‐2 tables that contain a zero entry but other tables are not modified in this way.

We conventionally estimate τ ^2^ and then treat this parameter as fixed and known when making inferences about the log odds ratio θ and hence inferences about the odds ratio, in the second stage. The conventional methodology is then straightforward (eg, Higgins et al^7^: Section 3.2). Although modifications of the usual random‐effects methodology have been proposed,(17, 18) we follow the most conventional random‐effects methods to provide results that can be compared to those from other models.

A wide variety of estimators of τ ^2^ are available.^19^ The DerSimonian and Laird^20^ estimator is so ubiquitous in application (because it is currently the default in many standard software packages for meta‐analysis) that we present results using this method, where we truncate this estimator to 0 in situations where it would otherwise be negative. The DerSimonian and Laird estimator matches a weighted sum of squares of the y_i_ to its expectation under model 1, where the weights are the reciprocals of the $s_{i}^{2}$. However, DerSimonian and Laird^20^ also considered the use of unweighted and likelihood‐based estimators of τ ^2^. The restricted maximum likelihood (REML) estimator(19, 21) uses a modification of the usual likelihood to compensate for the fact that maximum likelihood estimates of unknown variance components tend to be biased downwards. However, the standard theory of REML requires normally distributed outcome data. We consider REML to be the most suitable likelihood‐based implementation of model 1, so that the most appropriate comparison between the results from this model and those that follow is obtained by using REML.

Model 1 will already be very familiar to meta‐analysts and can be implemented in most standard meta‐analysis software packages. Here, we use the rma.uni command from the metafor R package for this purpose.

### 3.2 Model 2: the Simmonds and Higgins model

The next 6 models are less familiar, but closely related, generalised linear mixed models as described in the framework of Turner et al.^22^ Particular types of these models have subsequently been specifically proposed or implemented in software by others. We will attribute the models that follow to later authors and software packages, but much of what follows was anticipated by Turner et al.^22^ As we will explain, models 3 and 5 are special cases of model 6. Hence, many analysts are likely to prefer model 6 to these alternatives on the grounds that it avoids making unnecessary assumptions. We return to this issue later.

Simmonds and Higgins^10^ propose the model

$$\text{logit}(\pi_{ij})=\gamma_{i}+j\theta_{i},$$

where θ_i_∼N(θ,τ ^2^) and all θ_i_ are independent. The γ_i_ are fixed effects (unrelated constants) that describe the baseline risks of the event in each study.^10^ See Simmonds and Higgins' Equations (1) and (2) where they use a more general link function g(·) instead of logit(·), but here, we restrict ourselves to the logistic link because the odds ratio is used. An equivalent way to express this model, which is more directly related to the syntax used by the glmer function in R, is

(1) $$\text{logit}(\pi_{ij})=\gamma_{i}+j\theta +j\epsilon_{i},$$

where ϵ_i_∼N(0,τ ^2^) and all ϵ_i_ are independent. Since θ_i_=logit(π_i1_)−logit(π_i0_)=θ+ϵ_i_, we have that E[θ_i_]=θ and Var[θ_i_]=τ ^2^, so that θ and τ ^2^ continue to represent the summary log‐odds ratio, which can be transformed to the odds ratio scale, and between‐study variance, respectively. This also applies to the next 3 models.

#### 3.2.1 Fitting model 2

Models 2 to 7 are generalised linear mixed models which are usually fitted using maximum likelihood estimation. The maximisation of the likelihood required to fit these models is performed numerically and so may be fragile. We return to this issue below. As explained by Simmonds and Higgins,^10^ upon loading the lme4 package^23^ (version 1.1‐12 was used throughout), model 2 can be fitted in R using maximum likelihood estimation by specifying the logistic mixed‐effects regression model



glmer(cbind(event,n-event)~factor(study)+factor(treat)+(treat-1|study),data=thedata1,

family=binomial(link="logit")),





where “thedata1” is a dataframe containing columns of data as shown in the second set of column headings in Table 1. For this, and the models that follow, the regression coefficient associated with factor(treat) is the summary log‐odds ratio $\hat{\theta }$. The reported random‐effects variance is ${\hat{\tau }}^{2}$. Inferences using this model (for example, standard errors and confidence intervals), and all those that follow, are made using the asymptotic theory of maximum likelihood.

Because j is an indicator, “factor(treat)” can be replaced with “treat,” but we retain the syntax factor(treat) from Simmonds and Higgins^10^ to be consistent with their code. Also, an alternative parameterisation without an intercept can be used when calling glmer (by adding “−1” to the linear predictor), so that the coefficients associated with factor(study) are then immediately the estimates of γ_i_. Instead, including the intercept in the way shown means that (k−1) dummy covariates are associated with “factor(study).” Using this alternative parameterisation may result in slightly different estimates that agree to within numerical error.

### 3.3 Model 3: Simmonds and Higgins' model with random‐study specific effects

An alternative to using fixed effects for the γ_i_ in (1) is to instead assume that they are random‐effects γ_i_∼N(γ,σ ^2^). In this model, we assume that all γ_i_ and ϵ_i_ are independent. We will discuss the advantages and disadvantages of using fixed and random γ_i_ in Section 4.

#### 3.3.1 Fitting model 3

This model can be fitted as



glmer(cbind(event,n-event)~(1|study)+factor(treat)+(treat-1|study), data=thedata1,

family=binomial(link="logit")),





where the model intercept is γ. Now, there are 2 random‐effects variances in the model: the one associated with “(treat‐1|study)” continues to be τ ^2^ and the one associated with “(1|study)” is σ ^2^.

### 3.4 Model 4: a modified version of Simmonds and Higgins model

A modified version of the Simmonds and Higgins model (1) is the model

(2) $$\text{logit}(\pi_{ij})=\gamma_{i}+j\theta +z_{ij}\epsilon_{i}.$$

We include this model because it has been implemented in the metafor package.

In Equation 2, we can replace j θ with z_ij_ θ=(j−0.5)θ without changing the form of the model. This is because if we write logit(π_ij_)=γ_i_+z_ij_ θ+z_ij_ ϵ_i_, and then take $\gamma_{i}^{*}=\gamma_{i}-0.5\theta$, then we obtain (2) where γ_i_ is now $\gamma_{i}^{*}$. Hence, replacing j θ with z_ij_ θ in (2) is just a model reparameterisation. However, replacing j ϵ_i_ in (1) with z_ij_ ϵ_i_ to obtain (2) results in a different model, as we demonstrate by examining the implied covariance structure under these models in the paragraph immediately below. The subtle point is that the decision to use j or z_ij_ is immaterial when specifying the mean but not when describing the random effects. Hence, we modify (1) by replacing only the second j with z_ij_ to obtain model 4 as shown in Equation 2. Some readers may prefer model 2 to model 4, on the grounds that the heterogeneity component then more clearly represents heterogeneity in the treatment effects. However, we will see below that there are statistical reasons for preferring model 4; indeed, this is an important conclusion from this paper.

Models 2 and 4 appear very similar. The difference between these models is most clearly seen from the bivariate representation of the log odds of an event in the control and treatment groups of the ith study. From Equation 1, for model 2, we have

$$\left(\begin{array}{c} \text{logit}(\pi_{i0})\\ \text{logit}(\pi_{i1}) \end{array}\right)∼N\left(\left(\begin{array}{c} \gamma_{i}\\ \gamma_{i}+\theta \end{array}\right),\left(\begin{array}{cc} 0 & 0\\ 0 & \tau^{2} \end{array}\right)\right),$$

and from Equation 2, for model 4, we have

$$\left(\begin{array}{c} \text{logit}(\pi_{i0})\\ \text{logit}(\pi_{i1}) \end{array}\right)∼N\left(\left(\begin{array}{c} \gamma_{i}\\ \gamma_{i}+\theta \end{array}\right),\left(\begin{array}{cc} \tau^{2}/4 & -\tau^{2}/4\\ -\tau^{2}/4 & \tau^{2}/4 \end{array}\right)\right).$$

For both models, we have E[θ_i_]=θ and Var[θ_i_]=τ ^2^, but models 2 and 4 assume different bivariate structures.

#### 3.4.1 Fitting model 4

This model can be fitted using metafor as



rma.glmm(measure="OR", ai=A, bi=B, ci=C, di=D, data=thedata2, model="UM.FS"),





where “thedata2” is a dataframe with columns set out as in Table 2. Here “UM.FS” indicates an unconditional model and fixed‐study effects γ_i_. Metafor's alternative conditional (on the total number of events) model is presented as model 7 below. This model can also be fitted using glmer, for example, as



glmer(cbind(event,n-event)~factor(study)+factor(treat)+(treat12-1|study), data=thedata1, family=binomial(link="logit")).





In fact, the rma.glmm command calls glmer from the lme4 package to fit this and the next model.

### 3.5 Model 5: a modified version of Simmonds and Higgins model with random‐study specific effects

As in the Simmonds and Higgins model, an alternative to model 4 is to assume that γ_i_∼N(γ,σ ^2^) in (2). This model has also been implemented in the metafor package. As is the case for model 3, it is immaterial whether we write j θ or z_ij_ θ=(j−0.5)θ when specifying the model, because instead writing θ z_ij_ is a model reparameterisation where γ ^∗^=γ−0.5θ.

#### 3.5.1 Fitting model 5

This model can be fitted using metafor as 



rma.glmm(measure="OR", ai=A, bi=B, ci=C, di=D, data=thedata2, model="UM.RS"),





where “UM.RS” now indicates an unconditional model and random‐study effects γ_i_. As for the previous model, this can also be fitted using glmer which is called by the rma.glmm command, for example, as



glmer(cbind(event,n-event)~(1|study)+factor(treat)+(treat12-1|study), data=thedata1, family=binomial(link="logit")).





### 3.6 Model 6: the “Van Houwelingen bivariate” model

This model was originally proposed by Van Houwelingen et al^11^ and has also been presented by Stijnen et al.^12^ This model describes the joint distribution of the probability of an event in the treatment and control groups in each study. Specifically, this model assumes that

(3) $$\left(\begin{array}{c} \text{logit}(\pi_{i0})\\ \text{logit}(\pi_{i1}) \end{array}\right)∼N\left(\left(\begin{array}{c} \gamma \\ \gamma +\theta \end{array}\right),\left(\begin{array}{cc} \sigma_{0}^{2} & \rho \sigma_{0}\sigma_{1}\\ \rho \sigma_{0}\sigma_{1} & \sigma_{1}^{2} \end{array}\right)\right),$$

where we have reparameterised the mean differently to the previous descriptions of this model, so that θ continues to represent the log odds ratio. We now have

(4) $$\tau^{2}=\mathrm{Var}(\theta_{i})=\mathrm{Var}(\text{logit}(\pi_{i1})-\text{logit}(\pi_{i0}))=\sigma_{0}^{2}+\sigma_{1}^{2}-2\rho \sigma_{0}\sigma_{1}.$$

We can also present models 3 and 5 in the bivariate form shown in Equation 3. From Equations 1 and 2, with the assumptions that γ_i_∼N(γ,σ ^2^) and ϵ_i_∼N(0,τ ^2^), where these random effects are independent, we can write model 3 as

(5) $$\left(\begin{array}{c} \text{logit}(\pi_{i0})\\ \text{logit}(\pi_{i1}) \end{array}\right)∼N\left(\left(\begin{array}{c} \gamma \\ \gamma +\theta \end{array}\right),\left(\begin{array}{cc} \sigma^{2} & \sigma^{2}\\ \sigma^{2} & \sigma^{2}+\tau^{2} \end{array}\right)\right)$$

and model 5 as

(6) $$\left(\begin{array}{c} \text{logit}(\pi_{i0})\\ \text{logit}(\pi_{i1}) \end{array}\right)∼N\left(\left(\begin{array}{c} \gamma \\ \gamma +\theta \end{array}\right),\left(\begin{array}{cc} \sigma^{2}+\tau^{2}/4 & \sigma^{2}-\tau^{2}/4\\ \sigma^{2}-\tau^{2}/4 & \sigma^{2}+\tau^{2}/4 \end{array}\right)\right).$$

See also table V of Turner et al^22^ for (5) and (6), where these results are also given. From the bivariate representation of models 3 and 5 in (5) and (6), we can see that model 6 is a generalisation of both of these models. Compared to model 6, models 3 and 5 impose constraints on the covariance structure of the true underlying log odds of an event in the 2 treatment groups, whilst ensuring that τ ^2^=Var(θ_i_). Also from (5), we can easily derive that the true treatment effect in the ith study, logit(π_i1_)−logit(π_i0_), is independent of logit(π_i0_) in model 3. Furthermore, from (6), we can derive that logit(π_i1_)−logit(π_i0_) is independent of logit(π_i1_)+logit(π_i0_) in model 5. No such independence structure is imposed by model 6. The difference between models 2 and 4 is analogous to that between models 3 and 5.

#### 3.6.1 Fitting model 6

This model can be fitted as



glmer(cbind(event,n-event)~factor(treat)+(control+treat-1|study), data=thedata1,

family=binomial(link="logit")),





where we calculate the estimate of τ ^2^ from the estimated variance components associated with (control + treat‐1|study) as shown in Equation 4.

### 3.7 Model 7: the “hypergeometric‐normal” model

This model was also proposed by Van Houwelingen et al^11^ and Stijnen et al.^12^ Upon conditioning on the total number of events all marginal totals of the 2‐by‐2 tables are fixed. Then, each table can be specified in terms of the number of events in the treatment group, e_i1_, and we have

(7) $$P(E_{i1}=e_{i1}|\theta_{i})=\frac{\left(\begin{array}{c} n_{i1}\\ e_{i1} \end{array}\right)\left(\begin{array}{c} n_{i0}\\ t_{i}-e_{i1} \end{array}\right)\exp(\theta_{i}e_{i1})}{\sum_{j}\left(\begin{array}{c} n_{i1}\\ j \end{array}\right)\left(\begin{array}{c} n_{i0}\\ t_{i}-j \end{array}\right)\exp(\theta_{i}j)},$$

where we define the total number of events t_i_=e_i0_+e_i1_ and the summation j is over the values of e_i1_ that are permissible under the table marginal totals. Equation 7 is the noncentral hypergeometric distribution. If we take the log odds ratio in the ith study to be θ_i_=0, then all exponents in (7) are equal to 1 and, by Vandermonde's identity, the denominator of (7) becomes $\left(\begin{array}{c} n_{i0}+n_{i1}\\ t_{i} \end{array}\right)$. Hence, (7) reduces to the much more familiar (central) hypergeometric distribution when θ_i_=0. The noncentral hypergeometric distribution can therefore be conceptualised as “starting with the relative weights,” $\left(\begin{array}{c} n_{i1}\\ e_{i1} \end{array}\right)\left(\begin{array}{c} n_{i0}\\ t_{i}-e_{i1} \end{array}\right)$, of the probabilities allocated by the (central) hypergeometric distribution. If θ_i_>0, the noncentral hypergeometric distribution then gives further relative weight, and so probability, to large e_i1_ via the term $\exp\;(\theta_{i}e_{i1})$. If θ_i_<0, the noncentral hypergeometric distribution then gives less relative weight to large e_i1_ via this term.

Upon assuming that θ_i_∼N(θ,τ ^2^) and integrating out the random effects, we obtain the probability distribution of the ith table as

$$\int_{-\infty }^{\infty }P(E_{i1}=e_{i1}|x)\frac{1}{\tau }\phi ((x-\theta )/\tau )dx,$$

so that the likelihood is the product of terms of this type. The above integral is computationally more stable upon substituting z=(x−θ)/τ, to avoid division by a very small quantity when τ ^2^ is small. Model 7 is a mixed‐effects conditional logistic regression model.

#### 3.7.1 Fitting model 7

This model is implemented in metafor as 



rma.glmm(measure="OR", ai=A, bi=B, ci=C, di=D, data=thedata2, model="CM.EL"),





where “CM.EL” indicates conditional model and exact likelihood.

#### 3.7.2 An approximate version of model 7 when the event is rare

It is also possible to implement an approximate version of this model where it is assumed that the event is rare.^12^ First, note that if the event is rare, then the summation in the denominator of (7) is from j=0,1,2,⋯,t_i_. The result needed for the approximation is that, if y≪n, then $\left(\begin{array}{c} n\\ y \end{array}\right)\approx n^{y}/y!$ Then in the summation in the denominator of (7), we can approximate

$$\left(\begin{array}{c} n_{i1}\\ j \end{array}\right)\left(\begin{array}{c} n_{i0}\\ t_{i}-j \end{array}\right)\approx \frac{n_{i1}^{j}}{j!}\frac{n_{i0}^{t_{i}-j}}{(t_{i}-j)!}={\left(\frac{n_{i1}}{n_{i0}}\right)}^{j}\frac{n_{i0}^{t_{i}}t_{i}!}{j!(t_{i}-j)!t_{i}!}={\left(\frac{n_{i1}}{n_{i0}}\right)}^{j}\left(\begin{array}{c} t_{i}\\ j \end{array}\right)\frac{n_{i0}^{t_{i}}}{t_{i}!},$$

which, upon setting j=e_i1_, for the numerator of (7) gives

$$\left(\begin{array}{c} n_{i1}\\ e_{i1} \end{array}\right)\left(\begin{array}{c} n_{i0}\\ t_{i}-e_{i1} \end{array}\right)\approx {\left(\frac{n_{i1}}{n_{i0}}\right)}^{e_{i1}}\left(\begin{array}{c} t_{i}\\ e_{i1} \end{array}\right)\frac{n_{i0}^{t_{i}}}{t_{i}!}$$

so that (7) is approximately

$$P(E_{i1}=e_{i1}|\theta_{i})=\frac{\left(\begin{array}{c} t_{i}\\ e_{i1} \end{array}\right){\left(n_{i1}\exp(\theta_{i})/n_{i0}\right)}^{e_{i1}}}{{\text{∑}}_{j=0}^{t_{i}}\left(\begin{array}{c} t_{i}\\ j \end{array}\right){\left(n_{i1}\exp(\theta_{i})/n_{i0}\right)}^{j}}.$$

Next, we divide top and bottom by ${\left(1+n_{i1}\exp(\theta_{i})/n_{i0}\right)}^{t_{i}}$ and define $\pi_{i}=(n_{i1}\exp(\theta_{i})/n_{i0})/(1+n_{i1}\exp(\theta_{i})/n_{i0})$, so that

$$P(E_{i1}=e_{i1}|\theta_{i})=\frac{\left(\begin{array}{c} t_{i}\\ e_{i1} \end{array}\right)\pi_{i}^{e_{i1}}{\left(1-\pi_{i}\right)}^{t_{i}-e_{i1}}}{\overset{t_{i}}{\underset{j=0}{\text{∑}}}\left(\begin{array}{c} t_{i}\\ j \end{array}\right)\pi_{i}^{j}{\left(1-\pi_{i}\right)}^{t_{i}-j}}.$$

The denominator of this expression is the sum over all the probabilities of a Bin(t_i_,π_i_) distribution and so is one. The numerator is the probability of observing e_i1_ from this same distribution. Hence, if the event is rare, we can replace the hypergeometric density (7) with a much simpler binomial probability P(E_i1_=e_i1_|θ_i_) where E_i1_|θ_i_∼Bin(t_i_,π_i_). This is the same as expression (11) in Stijnen et al.^12^

We can write $\pi_{i}=\text{expit}(log(n_{i1}/n_{i0})+\theta_{i})$, where expit(·) is the inverse of the logit(·) function, so that the log odds of an event in the approximate model E_i1_|θ_i_∼Bin(t_i_,π_i_) is $log(n_{i1}/n_{i0})+\theta_{i}$. As we assume that θ_i_∼N(θ,τ ^2^), we can fit this approximate model as an intercept only logistic mixed‐effects regression model. Here, each study contributes a single binomial outcome where there are e_i1_ events in t_i_ trials, we specify an offset of $log(n_{i1}/n_{i0})$ and we include a random intercept in the model. The metafor package implements this approximate model using the syntax



rma.glmm(measure="OR", ai=A, bi=B, ci=C, di=D, data=thedata2, model="CM.AL"),





where “CM.AL” indicates conditional model, approximate likelihood. The intuition behind using a binomial approximation when the event is rare is because it is then of little consequence whether or not the model allows for the sampling to be performed with replacement. This type of approximation is used in many Cox regression and conditional logistic regression computer programs to avoid more numerically demanding exact likelihoods.

#### 3.7.3 Presenting a random‐effects implementation of the Peto method as an approximate version of model 7

Using the noncentral hypergeometric distribution is challenging numerically, and so it is desirable to have approximate methods that are numerically simpler to implement and so will be more robust. When the event is rare, then the approach suggested in the previous section can be used for this purpose. However, when the event is more common the large table entries make the computation even more challenging and another method is needed. In this section, we propose a method that can be used as a “sanity check,” to ensure that the results from model 7 are reliable.

The pooled log odds ratio using the Peto method^24^ is a 1‐step estimate of the log odds ratio, where we assume both the noncentral hypergeometric distribution (7) and a common‐effect model and take a single iteration using Fisher scoring method^25^ starting at θ=0. This means that the Peto method implements an approximate version of model 7 where τ ^2^=0. We however adopt a random‐effects approach.

To include random effects in a computationally straightforward way, we can take the study specific estimated Peto log odds ratios to represent the within‐study information contained in the noncentral hypergeometric distributions in (7). These estimated log odds ratios can be obtained using the escalc function and measure=“PETO.” Then using normal approximations for these estimated Peto log odds ratios, we can fit an approximate version of model 7 using the conventional random‐effects model (model 1) and the estimated Peto log odds ratios as outcome data. Maximum likelihood estimation is used when fitting model 7, and so we should estimate τ ^2^ by maximum likelihood when using the conventional model in this way to most accurately reproduce the results from model 7. This is easily implemented by taking using method=“ML” when using the rma.uni function to fit the standard random‐effects model.

The advantage of this approximate approach is that, unlike when fitting model 7 directly, the numerical methods required are very robust and convergence difficulties are rare. This approximate approach for fitting model 7 is especially useful to supplement simulation study results in situations where the estimation of this model fails, as we explain below. In applied work, more generally, we suggest that fitting the conventional random‐effects model to the estimated Peto log odds ratios, using maximum likelihood estimation for τ ^2^, is a practical way to assess if the sometimes numerically fragile results from model 7 are correct. If the numerical algorithms are successful and the estimated effect is not very large then these approximate inferences should be reasonably similar to those from model 7.

## 4 PROPERTIES OF THE STATISTICAL MODELS

Before we examine the implications of these statistical models empirically and via simulation studies, we discuss the main issues and concerns that relate to them.

Model 1, the conventional random‐effects model, has the advantages of being already familiar to meta‐analysts and very simple. However, the accuracy of the within‐study normality assumptions is often poor in practice which makes this approach open to criticism. The main reason for including this model is to see whether or not generalised linear mixed models, that avoid this type of normal approximation, can result in any notable improvement. Model 1 provides the current standard practice to compare the other models to.

Models 2 and 4 are conceptually very similar and both present a difficulty associated with using fixed effects for the γ_i_.^26^ This is because there is then a separate parameter γ_i_ for each study, so that if there are k studies, then there are k+2 parameters to estimate (the γ_i_, θ and τ ^2^). Each study provides 2 observations: the number of treatment and control group events, so that we have 2k observations to estimate these k+2 parameters. The model is therefore identifiable if k≥2, but the statistical issue is that the number of parameters increases at the same rate (ie, linearly) as the number of studies. Hence, as the sample size (the number of studies) becomes large, the number of parameters to estimate also becomes large. The asymptotic theory of maximum likelihood requires that the model, and so the number of parameters, is fixed as the sample size tends towards infinity, and this is not the case here (unless the number of studies is held fixed and the number of patients in them is allowed to tend towards infinity). Models 2 and 4 therefore do not satisfy the regularity conditions required for maximum likelihood estimation to possess its usual good properties. This suggests that any form of good classical estimation for these 2 models is likely to be challenging. Models 2 and 4 closely resemble models for Bayesian random‐effects models for meta‐analysis,^27^ which may have inspired the development of these classical models.

Models 3, 5, and 6 are also conceptually similar, and as explained above, model 6 is a generalisation of both of these other models. These models avoid the difficulty presented by models 2 and 4 because the number of parameters is fixed for all k. However, this is at the cost of making a distributional assumption about the control group rates; as shown in Section 3.6, models 3, 5, and 6 assume that the logit(π_i0_) are normally distributed. Then, as Senn^28^ points out, bias may result from the recovery of intertrial information. Compared to models 2 and 4, models 3, 5, and 6 can be thought of as solving a problem by introducing another. For example, we will see in our empirical investigation in Section 5 that “double‐zero” (no events) studies contribute information about θ in models 3, 5, and 6 via intertrial information.

Finally, the hypergeometric‐normal model (model 7) requires conditioning on the total numbers of events. The statistical principles that justify this conditioning are subtle, but an accessible account is given by Choi et al.^29^ As they point out, “opponents of conditioning argue that we cannot condition on the observed success total since it is not a perfect ancillary statistic  …  proponents argue that per the Conditionality Principle, the inference should be made by conditioning on the observed success totals which are approximately ancillary with little loss of information.” Choi et al also state that the Ancillarity Principle that is used to justify conditioning on an ancillary statistics is “generally less accepted than the idea of using a marginal density or likelihood based on a sufficient statistic.” Model 7 could therefore be criticised by those who do not accept the Ancillarity Principle or more practically on the grounds that some information is lost when using this model. However, those who might object to model 7 on these grounds would presumably also object to other closely related and well‐established statistical methods, such as Fisher exact test.

A further concern that accompanies the use of generalised linear mixed models, and therefore models 2 to 7, is that numerical maximisation of the nonnormal likelihood is needed to perform the estimation. This maximisation may be fragile. Furthermore, the random effects must be integrated out of the likelihood numerically, and this is computationally intensive and can be slow. When using the lme4 and metafor packages, we must choose a value for the variable “nAGQ,” the number of points per axis for evaluating the adaptive Gauss‐Hermite approximation to the log‐likelihood. The default is 1 when using lme4, which corresponds to the Laplacian approximation. However, the default is 7 when using the metafor package to fit models 4 and 5. To fairly compare results using models fitted using these packages, the same criterion for determining nAGQ should be used. We made the pragmatic decision to use the metafor default of “nAGQ=7” for generalised mixed models with 1 random effect (models 2, 4, and 7) and the lme4 default of “nAGQ=1” for models with 2 random effects (models 3, 5, and 6). This decision was made to reduce the computational demands of the simulation study, so that the fitting of many models that require integrating out 2 random effects from the likelihood is not computationally prohibitive. We return to this issue in the discussion. There is another tension between the defaults of the lme4 and metafor packages, in that the latter package's default when calling rma.glmm is to drop any studies where no event occurs prior to performing the analysis (“drop00=TRUE”). However, unless these studies are directly removed by the analyst, the glmer command in the lme4 package includes these studies in the model fitting. Since studies where no events occur contain very little information about relative treatment effects, such as the odds ratio, this difference between the default behaviours of the rma.glmm and glmer functions is not likely to have important consequences. However, we return to this issue in Sections 5 and 6 where we see that there are some more subtle issues relating to this point. The main observation is that all models are open to some form of criticism. The empirical and simulation studies that follow will examine whether or not these concerns have serious consequences in practice.

## 5 THE IMPLICATIONS OF USING THE STATISTICAL MODELS IN THE CONTEXT OF A COCHRANE REVIEW: ANTIBIOTICS FOR PREVENTING COMPLICATIONS IN CHILDREN WITH MEASLES

To explore the practical consequences of the modelling used, we conducted an empirical investigation using a Cochrane Review. The review Antibiotics for Preventing Complications in Children With Measles 30 was selected for this purpose because the odds ratio was used as the outcome measure, and this review was considered fairly typical of what is likely to be encountered in practice: The review includes multiple outcomes of interest, where relatively small numbers of studies contribute to each meta‐analysis. In this empirical evaluation, we examined only the primary analyses (1.1‐1.7). These analyses consist of comparing antibiotics to placebo or no antibiotic for different binary adverse events. The review also contained 23 further analyses, but these either describe baseline patient characteristics or sensitivity analyses. An unusual aspect of this review is the heterogeneous nature of the contexts in which the trials were performed: One study was conducted in India in the 1960s, another was conducted in West Africa in 2006, and the other 5 were performed in Glasgow, London and or New York^30^ between 1939 and 1954.

### 5.1 Description of data

The binary outcomes of interest in the review are (1) the development of pneumonia, (2) the development of diarrhoea, (3) the development of conjunctivitis, (4) the development of otitis media, (5) the development of croup, (6) the development of tonsillitis, and (7) death. Some of the included studies did not report results on all outcomes, so that the analyses contain differing numbers of studies. The data were taken directly from the Cochrane Review and are shown in Table 3, set out as in Table 2. The order in which the studies are listed in this table is different across outcomes; this was also the case in the Cochrane Review, and we retain the outcome specific ordering of treatments, so that Table 3 can more easily be seen to be the data reported in the Cochrane Review.

**Table 3:** Datasets used in empirical investigation, taken from Antibiotics for Preventing Complications in Children With Measles.^30^ Format matches that of Table 2

Outcome	Study	A	B	C	D
1 (pneumonia)	Karelitz, 1954	1	155	12	69
	Karelitz, 1951	0	89	3	40
	Garly, 2006	1	43	6	32
	Prasad, 1967	13	64	27	53
	Hogarth, 1939	2	157	5	165
	Anderson, 1939	4	43	6	43
	Gibel, 1942	6	76	0	148
2 (diarrhoea)	Anderson, 1939	5	45	12	38
	Garly, 2006	2	42	5	32
	Hogarth, 1939	8	151	7	163
	Karelitz, 1954	0	175	1	80
3 (conjunctivitis)	Garly, 2006	11	33	17	20
	Karelitz, 1951	0	88	0	43
4 (otitis media)	Anderson, 1939	3	57	7	52
	Garly, 2006	1	43	2	35
	Gibel, 1942	0	195	0	180
	Hogarth, 1939	5	154	12	158
	Karelitz, 1951	1	85	0	38
5 (croup)	Karelitz, 1951	0	87	1	42
6 (tonsillitis)	Anderson, 1939	0	63	8	54
	Karelitz, 1951	0	88	1	42
7 (death)	Anderson, 1939	3	60	1	61
	Garly, 2006	0	44	0	37
	Gibel, 1942	1	199	0	201
	Hogarth, 1939	0	159	0	170
	Karelitz, 1951	0	89	0	43
	Karelitz, 1954	0	175	0	81
	Prasad, 1967	0	78	0	80

An immediate observation from Table 3 is that only 3 of the outcomes include 3 or more studies where an event occurs. Although the final outcome, death, is included in all 7 studies, death only occurs in 2 of these. Studies with no events provide very little information about the odds ratio and are often discarded in analysis, so for some outcomes there are effectively just 1 or 2 studies that contribute to their analyses. The generalised linear mixed models that we have considered are, at best, extremely weakly identifiable when k=2. Hence, estimation issues, and even failures, are almost inevitable in these circumstances. We therefore did not attempt to fit our models to 4 of the outcomes (3, 5, 6, and 7). In general, we cannot recommend that applied analysts attempt this for models 2 to 7 unless there are 3 or more studies where an event occurs. This means that we only apply our methods to 3 of the outcomes (1, 2, and 4). Although this is perhaps disappointing, this is probably typical of what is possible in practice. Of the 3 outcomes that we apply our methods to, there is a further issue for outcome 4, where there is a single study where no event occurs. This study did not contribute to the corresponding analysis in the Cochrane Review on the grounds that the corresponding odds ratio is not estimable; as noted above, the default behaviour of rma.glmm is to remove these studies. We perform analyses for this outcome both including and excluding this study to assess the impact of this decision. We report the treatment effect estimates in terms of log odds ratios as set out in Section 3. The Cochrane Review presents results in terms of the odds ratio but the results are easily transformed from our scale to theirs. The Cochrane Review pooled the results using a Mantel‐Haenszel random‐effects method that we do not consider here; we instead use model 1 to provide a conventional analysis to compare other results to. However, the Mantel‐Haenszel method provides results that are in good agreement with ours. Each outcome is undesirable, and we use log odds ratios to compare antibiotic to either placebo or no antibiotic, where a negative estimate of treatment effect $\hat{\theta }$ indicates a benefit of antibiotics.

### 5.2 Estimation difficulties

Two minor estimation difficulties were initially encountered when performing the analyses. Firstly, for model 3 and the first outcome (pneumonia) an artificially small standard error for $\hat{\theta }$ was obtained. This was corrected by increasing the number of function evaluations allowed. Secondly, for model 7 and outcome 4, we changed the defaults to obtain convergence. Specifically, we increased the maximum number of iterations to 20 000, increased the relative tolerance to 0.0001, and used the more robust Nelder‐Mead maximisation algorithm. Hence, when fitting model 7 to outcome 4, we called rma.glmm using syntax of the form



rma.glmm(measure="OR", ai=A, bi=B, ci=C, di=D, data=thedata2, model="CM.EL", control=list(optCtrl=list(

maxit=20000, reltol=0.0001), optmethod="Nelder-Mead")).





We also specified nAGQ=7 when fitting model 7 because this model involves a single random effect (as explained in Section 4). However, this is just used in the computation when applying the approximate method in Section 3.7.2 to obtain starting values for the numerical maximisation and so does not control the number of quadrature points used when fitting this model using the metafor package. We explored the use of various alternatives to the current default of “hessianCtrl=list(16),” to try to obtain more stable standard errors but found that this default performed well and so retained it.

### 5.3 Results

The estimates of θ and τ ^2^ obtained under each model for all 3 outcomes analysed are shown in Table 4. Here, “outcome 4b” indicates the fourth outcome (otitis media) where the double‐zero study was removed prior to analysis. The standard errors of $\hat{\theta }$ are shown in parentheses. For all outcomes, the conclusions from all models are in broad agreement. However, there are also some noteworthy differences between the point estimates. For example, for outcome 1 (pneumonia) models 2 and 3 result in slightly greater estimated treatment effects and for outcome 2 (diarrhoea) there are also nonnegligible differences between the estimated effects. The estimates of τ ^2^ for outcomes 1 and 2, for which the outcome data are quite heterogeneous as noted in the Cochrane Review (I ^2^=63% and I ^2^=25%, respectively^30^), are sensitive to the model choice. However, estimates of τ ^2^ are not precise in small samples such as these. Hence, the magnitudes of the differences between the ${\hat{\tau }}^{2}$ for these outcomes are not surprising.

**Table 4:** Empirical investigation results. The top half of the table shows the treatment effect estimates $\hat{\theta }$ and their estimated standard errors in parentheses. The bottom half shows the estimates ${\hat{\tau }}^{2}$. Asterisks (*) denote cells where nondefault arguments have been used to obtain results. Outcome 4b represents the dataset from outcome 4 with its “double‐zero” study removed

	Outcome			
$\hat{\theta }$	1	2	4	4b
Model 1 (D&L)	−1.060(0.544)	−0.634(0.426)	−0.787(0.390)	−0.815(0.397)
Model 1 (REML)	−1.060(0.628)	−0.658(0.459)	−0.787(0.390)	−0.815(0.397)
Model 2	−1.236(0.782)	−0.599(0.345)	−0.803(0.396)	−0.803(0.396)
Model 3	−1.241(0.685)*	−0.683(0.433)	−0.815(0.396)	−0.878(0.396)
Model 4	−1.024(0.708)	−0.629(0.402)	−0.803(0.396)	−0.803(0.396)
Model 5	−1.071(0.717)	−0.673(0.413)	−0.815(0.396)	−0.878(0.396)
Model 6	−1.056(0.738)	−0.533(0.533)	−0.505(0.743)	−0.856(0.435)
Model 7	−1.143(0.888)	−0.635(0.416)	−0.793(0.395)*	−0.793(0.395)*
	**Outcome**			
${\hat{\tau }}^{2}$	1	2	4	4b
Model 1 (D&L)	1.154	0.183	0	0
Model 1 (REML)	1.785	0.275	0	0
Model 2	3.224	0	0	0
Model 3	2.311*	0.105	0	0
Model 4	2.664	0.085	0	0
Model 5	2.791	0.119	0	0
Model 6	2.789	0.071	0.197	0.004
Model 7	4.341	0.099	0.001*	0.001*

Abbreviation: REML, restricted maximum likelihood.

The most interesting observations are for outcome 4 (otitis media), where we compare the results where we do, and do not, remove the study where no events occurred. For model 1, the removal of the double‐zero study results in a point estimate further from the null, as should be expected. Models 2, 4, and 7 are robust to the choice of whether or not we include this study. This is obviously inevitable for model 7 because conditioning on no events results in a degenerate distribution that does not depend on the model parameters and so the double‐zero study does not contribute to the likelihood. This observation is, albeit less obviously, also inevitable for models 2 and 4. This is because double‐zero studies provide strong evidence that their study‐specific fixed γ_i_s are large and negative but otherwise provide negligible information about any other model parameter.

However, the results from models 3, 5, and 6 are sensitive to the decision of whether or not to include the double‐zero study. This is also as expected, because these models assume that the control group event rates are exchangeable, so that this study provides information about all model parameters via the recovery of intertrial information. With the exception of model 6 when including the double‐zero study (for which ${\hat{\tau }}^{2}=0.197$), all estimates of τ ^2^ are either 0 or very small, this is, consistent with the I ^2^=0 statistic for this outcome from the Cochrane Review.^30^ Hence, it would seem that model 6's estimate of τ ^2^, for the full dataset, is incongruous with the others. In the notation of Equation 3, model 6 provides ${\hat{\sigma }}_{0}^{2}=2.764$, ${\hat{\sigma }}_{1}^{2}=1.485$, and $\hat{\rho }=1$. Substituting these estimates into Equation 4 gives ${\hat{\tau }}^{2}=0.197$. However, upon removing the double‐zero study, we instead obtain ${\hat{\sigma }}_{0}^{2}=0.161$, ${\hat{\sigma }}_{1}^{2}=0.114$, and $\hat{\rho }=1$, which from Equation 4, we obtain ${\hat{\tau }}^{2}=0.004$. The most substantive difference between these estimated covariance structures is that the exclusion of the double‐zero study results in a markedly smaller ${\hat{\sigma }}_{0}^{2}$. This can be explained because the exclusion of this study drastically reduces the variability in the γ_i_, because we have removed the study where there is strong evidence that its γ_i_ is large and negative, and so ${\hat{\sigma }}_{0}^{2}$ is smaller upon omitting this study. Issues associated with the recovery of intertrial information for models 3, 5, and 6 are exemplified by the case where a decision is made regarding whether or not to include studies where no events occur.

The estimates of τ ^2^ using the “Peto approximation,” as described in Section 3.7.3, are ${\hat{\tau }}^{2}=1.949,0.069,0$, and 0. The corresponding estimates of θ, with standard errors in parentheses, are $\hat{\theta }=-0.914(0.595),-0.614(0.363),-0.747(0.359)$ and −0.769(0.365). Comparing these results to those for model 7 in Table 4, we can see that the point estimates from this approximation can provide a useful, albeit rough, check that the results from this model are correct.

The main conclusions from this empirical investigation are as follows. Firstly, there were only sufficient numbers of studies in 3 out of 7 analyses for the more sophisticated models to be advocated. We suspect this is likely to be fairly representative of other applications. Hence, the usefulness of the more sophisticated models 2 to 7 may be curtailed for this reason. In fact, obtaining accurate inference using the conventional model 1 is also very challenging when there are just a few studies^31^ but the numerical algorithms can be expected to be much more robust when using this simple model. Hence, there are fewer concerns about the stability of the numerical methods required when using the conventional model when there are very few studies, as is commonly the case. Secondly, this empirical investigation shows that inferences may be sensitive to modelling choice so sensitivity analysis is an attractive option. Thirdly, by considering the analyses of outcome 4, both including and excluding the double‐zero study, we have further proof of concept that models 3, 5, and 6 can recover intertrial information. This suggests that the decision of whether or not to include studies where no events occur should be considered carefully when using these models in application; sensitivity analysis to this decision when using these models are therefore likely to be desirable. If sensitivity analyses are not to be considered, then we suggest that it is better to exclude double‐zero studies when fitting models 3, 5, and 6 on the grounds that otherwise the recovery of intertrial information from these studies would be a source of considerable concern. Finally, the conventional random‐effects model has provided results that are in good agreement with the results from the more sophisticated models 2 to 7 which, given the first model's wide applicability and simplicity, suggests that the conventional approach retains its usefulness in application. We will explore this issue in more detail in the simulation study.

### 5.4 Additional real examples

In the Supporting Information, we explore 2 additional real examples that have different properties to those in the Cochrane Review described above. The first of these examples includes 45 studies. Here, because there are so many studies, the between‐study variance is better identified and all models result in the conclusion that there is no evidence of a treatment effect. However, point estimates of both θ and τ ^2^ are somewhat sensitive to the model used, and we conclude that sensitivity analyses retain their usefulness even in meta‐analyses with large numbers of studies.

The second additional example includes 17 studies and no events occur in 5 of these. The implications of the decision of whether or not to exclude the double‐zero studies was found to be similar to those for outcome 4 (otitis media) in our main example. In particular, models 2, 4, and 7 were not sensitive to this decision for the reasons given above. Models 3, 5, and 6 were sensitive to this decision because they recover intertrial information. However, these 3 models were not especially sensitive to this decision, despite the fact that a more substantial number of double‐zero studies contribute to this meta‐analysis. Further work is needed to better understand the conditions under which models 3, 5, and 6 are very sensitive to the decision of whether or not to include double‐zero studies.

Another observation is that for models 3 and 5, artificially small standard errors for $\hat{\theta }$ were initially obtained for the second additional example upon removing the 5 double‐zero studies. This was corrected by increasing the number of function evaluations allowed, as in Section 5.2 for model 3 and the first outcome. This suggests that obtaining reliable standard errors may be challenging when using generalised linear mixed models for meta‐analysis in practice. We return to this issue in the simulation study.

## 6 SIMULATION STUDY

The empirical investigation above is valuable but a simulation study was also performed to better understand the implications of using each of the models. We performed the simulation study over a range of settings designed to include favourable and unfavourable situations for different models. We focussed on 4 main issues: estimation failure, the bias and precision of the estimates of θ and τ ^2^, and the coverage of nominal 95% confidence intervals for θ. This means that we focus on the bias and precision of the estimated log odds ratio, rather than the odds ratio directly, but this is appropriate because all inference is performed on the log odds ratio scale. Nominal 95% confidence intervals from all models are only approximate, even for the more sophisticated models 2 to 7. This is because inference from these models is based on the asymptotic theory of maximum likelihood. Hence, some departure from 95% coverage was anticipated when using all models. Kuss^15^ also explores the use of generalised linear mixed models in a simulation study, but here we explore in detail how variations of this type of model compare to each other. Thomas et al^32^ also examine 2 of the generalised linear mixed models that we consider in a simulation study (our models 4 and 5) and conclude that the results using these models are “not importantly different.” We will see below that this is also the case in our simulation study; hence, our findings are in agreement with this previous finding.

### 6.1 Simulation study design

We explored 15 simulation settings, each with 2 values of θ=0,log(2), so that results both under and away from the null were obtained. However, other than to provide a different location of the estimated effects, we did not expect the value of θ to greatly influence our conclusions. This intuition was for the most part confirmed and so (with the exception of estimation failure rates) we present the results for θ=0 in tables in the main paper and θ=log(2) in tables in the Supporting Information. One thousand simulated datasets were produced for each setting and value of θ, so that in total, 30 000 simulated datasets were produced. Different random seeds were used for each setting and value of θ, but all models were fitted to the same simulated datasets.

#### 6.1.1 The simulation study defaults

We initially considered the case where there are k=10 studies, where the log odds of an event in the control group were simulated from L O_c_∼N(logit(0.2),0.3^2^), the number of patients N in the treatment groups were simulated from a discrete uniform distribution from 50 to 500, the number of patients in the control groups are also N(so that the 2 treatment arms in all studies contain the same number of patients), and we used a moderate τ ^2^=0.024. These settings provide our defaults. There are very many possibilities to explore, so to make the simulation study computationally feasible, we changed just 1 (or, in the case of setting 15, two) of these defaults when considering other possibilities.

#### 6.1.2 The event probabilities

In 10 of the simulation settings (1 to 7 and 10 to 12), we simulated the log odds of an event in the control groups using our default of L O_c_∼N(logit(0.2),0.3^2^), so that the majority (96%) of control group probabilities fall within the range [0.1, 0.3]. Hence, we simulate control group events that are unlikely to occur but are not rare. In setting 8, we simulated L O_c_∼N(logit(0.05),0.3^2^), to explore the implications of the event being rarer. In setting 9, we simulated L O_c_∼N(logit(0.01),0.3^2^), to explore the implications of the event being very rare. In setting 13, we simulated the probability of an event in the control group from a uniform distribution from 0.1 to 0.3, so that models 3, 5, and 6 that make normal distributional assumptions about the control group log odds are misspecified. In setting 14, we simulated the average (across both groups) log odds of an event as L O_a_∼N(logit(0.2),0.3^2^), so that data are then simulated under models 4 and 5 (instead of models 2 and 3). In setting 15, we simulated L O_c_∼N(logit(0.5),0.3^2^), so that events and nonevents are approximately equally likely in control groups. As explained below, in setting 15, we explore the implications of extreme between‐study heterogeneity. By taking the control group event probabilities to be around 0.5, and then allowing the treatment group probabilities to take a wide‐range values (see below), we are able to invoke very considerable between‐study variation in the comparative event rates across studies.

In all but setting 14, we then simulated the log odds of an event in the treatment group as L O_t_=L O_c_+θ+τ Z, where Z∼N(0,1). For setting 14, we also simulated the treatment effects as θ+τ Z, but we added and subtracted half of this to the treatment and control group event log odds, respectively. Log odds were converted to probabilities so that binary data could be simulated using binomial distributions.

#### 6.1.3 The numbers of patients

In 11 of the simulation settings (1 to 6, 8 to 9, and 13 to 15), we simulated the number of patients N in the treatment group using our default distribution, a discrete uniform distribution from 50 to 500, and we also took the same number of patients in the control groups. This produces a range of realistic study sizes where the studies are neither very small or inordinately large, and where the 2 treatment groups are of equal size. In setting 7, we instead simulated N from a discrete uniform distribution from 10 to 100, to explore the impact of smaller studies. It is not uncommon for meta‐analyses to include only small studies, so setting 7 represents a situation that is quite likely to arise in practice. The normal approximations required by model 1 are really quite crude in this setting.

In the remaining 3 simulation settings (10 to 12), we explored the use of unequal group sizes (but otherwise used our defaults). In setting 10, we set the control group size to be half that of the treatment group. This reflects the notion that 2 closely related treatment groups might be combined to form a single treatment group. In this setting, we therefore took the number of patients in all the control groups to be N/2, where halves were rounded to a whole number. In setting 11, we took the number of patients in 50% (k/2=5) of the control groups to be N, the same as in the treatment group, but in the other 50% of the control groups, we took the number of patients in the control group to be N/2. In this setting, studies were allocated to these 2 types of study on the basis of the order in which they were simulated, so that there is no association between the control group size and the outcome data. We did not expect this setting to result in bias for the estimated effect when using any model.

However, in setting 12, we ranked the k=10 true probabilities of an event in the control groups and took those whose rank is less than equal to k/2=5 to have the same number of N patients as in the corresponding treatment group. The remaining 5 studies were instead taken to have N/2 patients in the control group. This setting was explored as it was thought to have the potential to result in bias in models 3, 5, and 6. This is because the distributional assumptions for the control group event probabilities, combined with an association between event probabilities and the control group size, were thought to be good conditions under which the recovery of intertrial information might result in bias when using these models.

#### 6.1.4 A typical study within‐study variance

The within‐study variance for an estimated log odds ratio is a function of the group sizes and the event probabilities. Having specified the way in which we determine these quantities, we can determine a typical within‐study variance. This enables us to better understand the implications of the simulation study design.

Taking the typical number of patients in each treatment group to be 275, and a typical event probability to be 0.2, a representative within‐study variance for the resulting estimated log odds ratios is around 2/(275×0.2×0.8)≈0.05. This is similar to the typical within‐study variance of 0.056 from the scaled and truncated χ ^2^ distribution that has sometimes been used to simulate typical within‐study variances for log odds ratios.(33, 34, 35, 36) This observation indicates that the study sizes and probabilities of an event are reasonably consistent with what is typically observed in practice.

#### 6.1.5 The number of studies

In 12 of the simulation settings (1 to 3 and 7 to 15), we simulate using our default of k=10 studies. This produces meta‐analyses where models are well identified but the number of studies is not unrealistically large. In the other settings we explore the use of k=3,5,20, to explore the implications of the number of studies. We consider k≥3 to investigate situations where all models are identifiable. Each study provides the treatment and control event rates, so that in total, a meta‐analysis has 2k estimates. Then, with k=3, we have 6 estimates available and all models are, albeit weakly, identifiable. We deliberately avoid the case where k=2 where models may be identifiable but estimation problems are very likely, as in the empirical evaluation in Section 5.

#### 6.1.6 The between‐study variance

In simulation settings 1 to 3, we simulate datasets with the above defaults and with τ ^2^=0.024,0,0.168, respectively. These values were used previously(35, 36) with the scaled and truncated χ ^2^ distribution described above to reflect I ^2^ statistics^37^ of 0.3, 0, and 0.75. Hence, these values were chosen to be consistent with previous simulation studies(35, 36) and to explore mild, no, and considerable between‐study variation, respectively. In simulation settings 4 to 14, we simulated data with our default τ ^2^=0.024, to explore how the methods perform when between‐study heterogeneity is present but is mild. We chose the moderate τ ^2^=0.024 as our default as we thought that random‐effects models are most likely to be applied in situations where between‐study heterogeneity is present but moderate.

The analysis of outcome 1 in Table 4 would seem to suggest that much larger values of τ ^2^ are possible in practice. A 95% confidence interval for τ ^2^ for this dataset, using the Q profile method,^19^ is (0.166, 20.492). Hence, there is very little information about this parameter for this example. However, in Table 3, we can see that studies provide markedly different comparative rates and it is of interest to explore extreme scenarios such as these. To investigate such a situation, in setting 15, we simulated data using τ ^2^=2. As explained above, we simulate L O_c_∼N(logit(0.5),0.3^2^) in this setting; taking control group event probabilities to be 0.5, this means that the majority (96%) of treatment event group probabilities will lie within (0.05, 0.95). This very wide range of treatment group probabilities means that studies with very markedly different treatment group event rates, and so comparative rates, will be commonplace in this setting. Setting 15 therefore explores the implications of extreme between‐study heterogeneity.

#### 6.1.7 Producing outcome data

Binary outcome data were simulated using binomial distributions with the resulting group sizes and event probabilities. For model 1, the metafor function escalc was used, with its defaults for handling zeroes, so that empirical log odds ratios and within‐study variances can be calculated and used in the conventional random‐effects model. For all other methods, the data were processed into the forms shown in Tables 1 and 2, so that models 2 to 7 can be fitted.

A summary of the 15 simulation settings is shown in Table 5, where departures from the defaults are shown in bold. In this table, ∼U and ∼N indicate that data were simulated from a uniform and a normal distribution, respectively. L O_c_ and P_c_ indicate the log odds of an event and the probability of an event in the control group, respectively. L O_a_ is the average log odds across both groups. N is the number of patients in the treatment group, which is usually also the number in the control group. k is the number of studies. NR indicates “Not Random,” which refers to the fact that in setting 12, the studies that have smaller control groups are not chosen at random.

**Table 5:** Simulation study design. All 15 settings were performed with θ=0 (results shown in tables in the main paper) and θ=log(2) (results shown in tables in the Supporting Information). One thousand simulated datasets were produced in each setting and true effect, so that in total, 30 000 datasets were produced. A different random seed was used for each setting and value of θ. All models were applied to the same datasets. This table provides an outline of the simulation study design; see Section 6 for full details. Departures from the defaults are shown in bold

Setting	k	τ ^2^	Treatment	Control	Baseline probability	Correct models
1	10	0.024	N∼U(50,500)	N	L O_c_∼N(logit(0.2),0.3^2^)	2, 3, 6
2	10	**0**	N∼U(50,500)	N	L O_c_∼N(logit(0.2),0.3^2^)	2, 3, 6
3	10	**0.168**	N∼U(50,500)	N	L O_c_∼N(logit(0.2),0.3^2^)	2, 3, 6
4	**3**	0.024	N∼U(50,500)	N	L O_c_∼N(logit(0.2),0.3^2^)	2, 3, 6
5	**5**	0.024	N∼U(50,500)	N	L O_c_∼N(logit(0.2),0.3^2^)	2, 3, 6
6	**20**	0.024	N∼U(50,500)	N	L O_c_∼N(logit(0.2),0.3^2^)	2, 3, 6
7	10	0.024	N∼U(**10**,**100**)	N	L O_c_∼N(logit(0.2),0.3^2^)	2, 3, 6
8	10	0.024	N∼U(50,500)	N	L O_c_∼N(logit(**0.05**),0.3^2^)	2, 3, 6
9	10	0.024	N∼U(50,500)	N	L O_c_∼N(logit(**0.01**),0.3^2^)	2, 3, 6
10	10	0.024	N∼U(50,500)	**N/2**	L O_c_∼N(logit(0.2),0.3^2^)	2, 3, 6
11	10	0.024	N∼U(50,500)	**N/2** **and** **N**	L O_c_∼N(logit(0.2),0.3^2^)	2, 3, 6
12	10	0.024	N∼U(50,500)	**N/2** **and** **N (NR)**	L O_c_∼N(logit(0.2),0.3^2^)	None
13	10	0.024	N∼U(50,500)	N	**P**_c_∼**U**(**0** **.** **1** **,** **0** **.** **3**)	2
14	10	0.024	N∼U(50,500)	N	**LO**_a_∼N(logit(0.2),0.3^2^)	4, 5, 6
15	10	**2**	N∼U(50,500)	N	L O_c_∼N(logit(**0.5**),0.3^2^)	2, 3, 6

### 6.2 The implications of our defaults when fitting models

With the exception of model 7 (the numerically challenging hypergeometric‐normal model), and also when correcting standard errors for models 3, 5, and 6 in setting 9 (see below), we used the defaults of the escalc and rma.uni commands (model 1), the glmer command (models 2, 3, and 6), and the rma.glmm command (models 4, 5, and 7), with our convention for the value of “nAGQ” explained above. That is, when an implementation of a model using the metafor package was available, we used it, but when this was not available, we used glmer from the lme4 package directly.

The impact of the default behaviour of rma.glmm, which removes any studies where no events occurred in either treatment group prior to analysis (which is not the default of the glmer function) is negligible in 14 settings: excluding setting 9 (very rare events), only 13 studies where no events occurred were simulated across the whole simulation study (7 studies in setting 7 and θ=0; 4 studies in setting 8 and θ=0; 2 studies in setting 7 and θ=log(2)). However, in setting 9, double‐zero studies were unusual but not rare: 494 studies were simulated when θ=0, and 205 studies were simulated when θ=log(2). We produced 1000 studies for each setting and value of θ, and setting 9 uses our default with k=10, so that these rates correspond to proportions of 494/10000=4.9% and 205/1000=2.1% of double‐zero studies.

The nonnegligible rates of double‐zero studies in setting 9 has implications for model 1. As explained above, we use the escalc command with its defaults to obtain outcome data for this model, which means that halves are added to all entries of tables that contain zeroes. Hence, double‐zero studies are included when fitting model 1 in this way but these studies are typically removed in practice, perhaps on the grounds that their odds ratio is considered to be “not estimable” as in the Cochrane Review. We have established that models 2, 4, and 7 are not sensitive to the decision of whether or not to include double‐zero studies. However, models 3, 5, and 6 are sensitive, and we have argued that it is probably better to exclude double‐zero studies when fitting them. These studies are excluded in our simulation study when fitting model 5 because we use the rma.glmm command with its defaults, but including them when using our defaults with the glmer command to fit models 3 and 6 is a cause of concern in setting 9. In Section 6.7, we will therefore investigate the implications of instead excluding double‐zero studies when fitting models 1, 3, and 6 in setting 9.

### 6.3 Simulation study results

We present the results in 3 sections, where we examine estimation failure, bias and precision, and the coverage probability of confidence intervals. We summarise the results for estimation failure across all simulation settings and θ in the main paper (all 30 000 simulated datasets), but for bias and coverage probability, we show the results for θ=0 in the main paper (15 000 simulated datasets) and the remaining results for θ=log(2) in the Supporting Information. The simulation study was timed for setting 1 and θ=0, using a computer containing an i7‐4790 processor and 16 gigabytes of RAM. Computation times for fitting the models to the 1000 datasets were 5 seconds (model 1, DerSimonian and Laird), 9 seconds (model 1, REML), 6 minutes (model 2), 2 minutes (model 3); 7 minutes (model 4), 10 minutes (model 5), 3 minutes (model 6), and 6 hours and 4 minutes (model 7). This indicates that models 1 and 7 are easily the least, and most, computationally demanding models to fit, respectively.

### 6.4 Estimation failure

As explained above, the numerical methods required when fitting generalised linear mixed models can be fragile. This is because the likelihood must be maximised numerically and this maximisation may fail. In this section, we quantify these difficulties and explain how we overcame these problems.

#### 6.4.1 Model 1

For model 1, the REML estimator failed to achieve convergence in only 11 out of the 30 000 simulated datasets and, because the estimating equation is of closed form, the DerSimonian and Laird estimator encountered no estimation failures. Although a skilled statistician could change the defaults and ultimately force convergence of the REML estimator, because the impact of such a small proportion of estimation failures is negligible these datasets were discarded when evaluating model 1 and REML.

#### 6.4.2 Models 2 to 6

For model 2, estimation failure was encountered in just a single dataset and model 3 encountered no failures at all. Similarly, models 4 and 5 encountered failures in 1 and no datasets, respectively, where the same single dataset resulted in estimation failures for multiple models. This dataset occurred in the fourth setting, where k=3 and ${\hat{\tau }}^{2}=0$, and the simulated outcome data in the 3 treatment groups were all identical to the data in the corresponding control groups. It is not surprising that small and highly homogenous datasets can result in the occasional estimation problems for this type of model. This is because there is relatively little information available about τ ^2^ when k is small, and it is challenging to estimate excess variance components when the data are completely homogenous. Model 6 encountered no estimation failures. Hence, the general picture from this simulation study is that, provided that k≥3, estimation failure is a very rare event in all models except model 7. The single dataset that caused estimation problems was discarded when evaluating the models that it resulted in estimation failure for, with negligible impact on the results.

As seen in Sections 5.2 and 5.4, artificially small standard errors for $\hat{\theta }$ can be obtained when fitting generalised linear mixed models. All reported standard errors for $\hat{\theta }$ from models 2 to 6 were therefore checked to assess whether or not they appeared to be reliable. With the exception of setting 9 (very rare events), all these standard errors seemed to be appropriate. However , in setting 9, artificially small standard errors for models 3, 5, and 6 were quite common when using the estimation defaults. For model 3, 74/1000 standard errors appeared to be artificially small for θ=0, and 60/1000 standard errors appeared to be artificially small for θ=log(2). For model 5, these rates were 52/1000 and 39/1000, and for model 6, these rates were 66/1000 and 71/1000, respectively. For models 3 and 5, all of these artificially small standard errors were corrected by increasing the number of function evaluations allowed, as in the real examples. However, this failed to improve matters for 2 simulated datasets when fitting model 6. A pragmatic decision was made for these 2 datasets, where we also used the standard error reported by model 5 for model 6. Ultimately, estimates and reliable standard errors could be obtained for all simulated datasets under models 2 to 6, except the single highly homogeneous dataset in the fourth setting described in the previous paragraph.

#### 6.4.3 Model 7

Model 7 is by far the most computationally challenging to fit, where estimation failure was fairly common across all simulation settings. For model 7, we changed the defaults as described in Section 5.2 for outcome 4 of the Cochrane Review. This resulted in substantially fewer estimation failures, where point estimates of θ and τ ^2^ were not obtained in 431/30000 (1.4%) datasets; standard errors for $\hat{\theta }$ were not obtained in 827/30000 (2.8%) datasets. However, there was the further difficulty in that some reported standard errors, although computed and provided, appeared to be unreliable because they differed substantially from those from other models, including the Peto approximation in Section 3.7.3. Most of these standard errors appeared to be clearly artificially low, but occasionally, a value that appeared to be too large was produced. On a single occasion a very grossly large standard error was reported by model 7 (17.5; the largest standard error reported by any other model was 0.09). Given that in around 1% of datasets estimates, but not standard errors, are obtained, it is not surprising that occasional unreliable standard errors will be computed when using model 7. This reinforces the suggestion in Section 3.7 that it is desirable to have approximate and robust methods for fitting model 7, to have a “sanity check” when reporting the results from this model.

A pragmatic approach for replicating the statistical expertise necessary to fit model 7 was adopted. We began by obtaining results using rma.glmm with the changes to the defaults described above. Then, any missing estimates or standard errors were replaced with those from the Peto approximation described in Section 3.7.3. Next, any reported standard errors that were less than 50% of that from the Peto approximation were replaced with those from this approximation. Finally, the grossly large standard error was also replaced with the standard error from this approximation. There was then a further issue because we found that in 41/30000 (0.1%) simulated datasets that the standard error reported by model 7 was more than twice that of the Peto approximation, and some of these will be erroneous. However, determining appropriate criterion to determine erroneously large standard errors is challenging and their impact on the simulation study is negligible. These 41 large standard errors from model 7 were therefore retained. In total, 992/30000 (3.3%) of the standard errors for model 7 were provided by the Peto approximation. By “supplementing” the results from model 7 with those from the Peto approximation, estimates and standard errors could be obtained from this model for all 30 000 simulated datasets.

### 6.5 Bias and precision of the estimates of θ and τ ^2^

In Table 6, we show the average of the estimates of θ and τ ^2^ in all 15 simulation settings and for θ=0. We also show the empirical standard errors of the estimates in parentheses. We show 2 sets of results for model 1, which show the results using the DerSimonian and Laird and REML estimators. The results for model 7 are indicated by model 7^∗^ in Table 6, to emphasise that these have been supplemented with results from the Peto approximation described in Section 3.7.3, as explained in Section 6.4.3. The Monte Carlo standard errors of the average estimates, and the empirical standard errors, in Table 6 can be obtained as explained by White.^38^ All Monte Carlo standard errors in Table 6 (of both the mean estimates and the empirical standard errors) in settings 1 to 8 and 10 to 14 are less than or equal to 0.005, to 3 decimal places. However, settings 9 (very rare events) and 15 (extreme between‐study heterogeneity) are more challenging situations. The maximum Monte Carlo standard errors in these settings is 0.010 and 0.014, respectively.

**Table 6:** Simulation study results. The top half of the table shows the mean estimate of the summary log odds ratio θ; empirical standard errors of the estimates are shown in parentheses. Monte Carlo standard errors of the mean estimates can be obtained as the empirical standard errors divided by the square root of 1000. The bottom half of the table shows the mean estimate of τ ^2^. The true value θ=0; results for θ=log(2) are shown in the Supporting Information. Model 7^∗^ indicates that inferences for model 7 have been supplemented with results from the Peto approximation described in Section 3.7.3, as explained in Section 6.3.3

	Model 1	Model 1	Model 2	Model 3	Model 4	Model 5	Model 6	Model 7^∗^
Setting	(D & L)	(REML)	(ML)	(ML)	(ML)	(ML)	(ML)	(ML)
1 (θ=0)	−0.001 (0.088)	−0.002 (0.088)	−0.002 (0.089)	−0.006 (0.088)	−0.001 (0.088)	−0.001 (0.088)	−0.006 (0.088)	−0.002 (0.088)
2 (θ=0)	−0.002 (0.070)	−0.002 (0.070)	−0.002 (0.071)	−0.004 (0.071)	−0.002 (0.071)	−0.002 (0.071)	−0.002 (0.072)	−0.002 (0.071)
3 (θ=0)	0.010 (0.153)	0.009 (0.154)	0.007 (0.156)	−0.002 (0.154)	0.010 (0.154)	0.013 (0.154)	−0.002 (0.155)	0.009 (0.155)
4 (θ=0)	0.002 (0.159)	0.001 (0.159)	0.003 (0.157)	−0.003 (0.159)	0.003 (0.157)	0.003 (0.157)	0.000 (0.162)	0.003 (0.157)
5 (θ=0)	−0.002 (0.119)	−0.002 (0.119)	−0.001 (0.119)	−0.005 (0.119)	−0.001 (0.119)	−0.001 (0.119)	−0.004 (0.121)	−0.001 (0.119)
6 (θ=0)	0.007 (0.060)	0.007 (0.060)	0.008 (0.060)	0.002 (0.060)	0.007 (0.060)	0.007 (0.060)	0.003 (0.060)	0.007 (0.060)
7 (θ=0)	0.010 (0.163)	0.011 (0.163)	0.011 (0.168)	0.001 (0.169)	0.009 (0.169)	0.010 (0.166)	0.004 (0.171)	0.009 (0.167)
8 (θ=0)	0.020 (0.137)	0.020 (0.137)	0.021 (0.141)	0.010 (0.142)	0.021 (0.141)	0.021 (0.141)	0.013 (0.144)	0.021 (0.141)
9 (θ=0)	0.003 (0.241)	0.002 (0.241)	−0.004 (0.292)	−0.035 (0.305)	−0.004 (0.289)	−0.001 (0.285)	−0.015 (0.324)	−0.006 (0.299)
10 (θ=0)	−0.006 (0.099)	−0.007 (0.099)	0.000 (0.100)	−0.006 (0.099)	−0.001 (0.100)	−0.001 (0.099)	−0.005 (0.101)	0.000 (0.100)
11 (θ=0)	0.001 (0.092)	0.001 (0.093)	0.004 (0.093)	−0.001 (0.092)	0.003 (0.093)	0.004 (0.092)	0.000 (0.093)	0.004 (0.093)
12 (θ=0)	0.002 (0.092)	0.002 (0.092)	0.006 (0.093)	0.017 (0.093)	0.005 (0.093)	0.017 (0.093)	0.017 (0.094)	0.005 (0.093)
13 (θ=0)	0.005 (0.087)	0.005 (0.087)	0.006 (0.087)	0.000 (0.087)	0.005 (0.087)	0.005 (0.087)	0.001 (0.089)	0.005 (0.087)
14 (θ=0)	−0.002 (0.090)	−0.002 (0.090)	−0.003 (0.091)	−0.006 (0.091)	−0.002 (0.091)	−0.002 (0.090)	−0.002 (0.092)	−0.002 (0.091)
15 (θ=0)	0.017 (0.439)	0.018 (0.443)	0.017 (0.451)	0.017 (0.451)	0.017 (0.446)	0.017 (0.442)	0.016 (0.451)	0.016 (0.448)
1 (τ ^2^=0.024)	0.026 (0.029)	0.026 (0.031)	0.006 (0.016)	0.020 (0.024)	0.020 (0.026)	0.020 (0.026)	0.023 (0.026)	0.020 (0.026)
2 (τ ^2^=0)	0.008 (0.016)	0.007 (0.015)	0.001 (0.005)	0.005 (0.013)	0.005 (0.012)	0.005 (0.012)	0.009 (0.014)	0.005 (0.012)
3 (τ ^2^=0.168)	0.160 (0.107)	0.165 (0.110)	0.115 (0.099)	0.147 (0.097)	0.144 (0.099)	0.143 (0.098)	0.148 (0.100)	0.145 (0.100)
4 (τ ^2^=0.024)	0.041 (0.071)	0.043 (0.082)	0.009 (0.033)	0.021 (0.049)	0.019 (0.044)	0.018 (0.042)	0.033 (0.056)	0.018 (0.044)
5 (τ ^2^=0.024)	0.030 (0.046)	0.031 (0.051)	0.008 (0.027)	0.021 (0.039)	0.019 (0.037)	0.018 (0.036)	0.028 (0.041)	0.019 (0.038)
6 (τ ^2^=0.024)	0.026 (0.022)	0.025 (0.022)	0.005 (0.012)	0.023 (0.020)	0.023 (0.021)	0.023 (0.021)	0.024 (0.021)	0.023 (0.021)
7 (τ ^2^=0.024)	0.048 (0.076)	0.045 (0.075)	0.004 (0.022)	0.036 (0.065)	0.039 (0.070)	0.038 (0.068)	0.062 (0.076)	0.039 (0.070)
8 (τ ^2^=0.024)	0.036 (0.057)	0.034 (0.058)	0.003 (0.019)	0.028 (0.047)	0.029 (0.052)	0.030 (0.055)	0.047 (0.058)	0.031 (0.056)
9 (τ ^2^=0.024)	0.024 (0.084)	0.026 (0.090)	0.013 (0.211)	0.077 (0.210)	0.077 (0.182)	0.094 (0.246)	0.148 (0.274)	0.130 (0.780)
10 (τ ^2^=0.024)	0.028 (0.035)	0.027 (0.036)	0.001 (0.007)	0.021 (0.030)	0.017 (0.028)	0.022 (0.032)	0.027 (0.033)	0.021 (0.031)
11 (τ ^2^=0.024)	0.030 (0.035)	0.029 (0.036)	0.004 (0.013)	0.022 (0.029)	0.021 (0.030)	0.023 (0.031)	0.027 (0.031)	0.023 (0.031)
12 (τ ^2^=0.024)	0.029 (0.035)	0.029 (0.037)	0.004 (0.017)	0.023 (0.032)	0.021 (0.031)	0.023 (0.032)	0.027 (0.032)	0.023 (0.032)
13 (τ ^2^=0.024)	0.029 (0.031)	0.028 (0.032)	0.007 (0.017)	0.022 (0.028)	0.022 (0.028)	0.022 (0.028)	0.026 (0.028)	0.022 (0.028)
14 (τ ^2^=0.024)	0.029 (0.032)	0.028 (0.033)	0.007 (0.019)	0.018 (0.024)	0.022 (0.028)	0.022 (0.028)	0.025 (0.028)	0.022 (0.028)
15 (τ ^2^=2)	1.364 (0.602)	1.922 (0.923)	1.771 (0.906)	1.795 (0.903)	1.744 (0.853)	1.706 (0.828)	1.792 (0.907)	1.769 (0.908)

Abbreviation: REML, restricted maximum likelihood.

#### 6.5.1 Evidence of bias of estimates of θ

There is very little evidence of bias in the estimates of θ in Table 6. The only 2 settings where bias is strongly statistically significant (where average estimates are around 5 Monte Carlo standard errors from 0) is setting 8 (where the event is rarer but not very rare) and setting 12 (which was designed to facilitate the recovery of intertrial information and so produce bias in models 3, 5, and 6). However, in all instances, these biases are very small and unimportant. The slight bias for model 1 in setting 8 is possibly because the association between the log odds ratios and their within‐study variances, and so the study weights, is strongest when the event rate is low. The generalised linear mixed models do not in general appear to be very effective in removing this bias, which is perhaps disappointing. In setting 12, we observe bias exactly where we would expect to see it, in the models that use random effects to model the control group event rate (models 3, 5, and 6). This is further proof of concept of Senn's^28^ argument that bias may result from the recovery of intertrial information, but the results also indicate that this bias is very small and so is not a serious cause of concern.

The conclusion that setting 12 results in small and unimportant, but detectable, bias in $\hat{\theta }$ under models 3, 5, and 6 is confirmed by the results for θ=log(2) in the Supporting Information. Most of the other conclusions for θ=0 are confirmed when using θ=log(2) but there are 2 exceptions. Firstly, there was no evidence of bias in setting 8 when θ=log(2). Secondly, in setting 9 (very rare events), bias towards the null (of around 0.06, which is substantial compared to the true θ=log(2)≈0.69) was detected when fitting model 1. This is an evidence that the normal approximations used by this model are not sufficiently accurate in this setting. The other models were effective in removing this bias in setting 9. However, some of this bias from model 1 may be attributable from the inclusion of double‐zero studies by adding halves, and we investigate this in Section 6.7 below.

#### 6.5.2 Evidence of bias of estimates of τ ^2^

The situation for the bias in estimates of τ ^2^ is a little more complicated. The most serious issue is for model 2, to which we devote an entire subsection below.

When τ ^2^ is moderate or 0, model 1 results in positively biased estimates of this parameter. This makes intuitive sense as point estimates that would otherwise be negative are truncated to 0. For example, under the assumptions of model 1, the DerSimonian and Laird estimator is unbiased before truncation. Hence, if model 1 describes the data well, then positive bias after truncation is inevitable. However, when τ ^2^ is large (setting 3), we observe slight negative bias in the DerSimonian and Laird estimator, and when τ ^2^ is extremely large (setting 15), we observe severe negative bias. These findings are perhaps counterintuitive. However, truncation occurs less often when τ ^2^ is large, so we should expect less positive bias in setting 3 and, in particular, setting 15. Furthermore, the conventional random‐effects model only provides an approximation; in particular, it ignores the association between the study‐specific estimates and their within‐study variances, so that negative bias is possible. This is because the most extreme (by chance) outcome data y_i_ result in smaller table entries, and hence large $s_{i}^{2}$, and so artificially small weights in the DerSimonian and Laird estimation method. Sidik and Jonkman^39^ also found negative bias in the DerSimonian and Laird estimator when simulating binary outcome data and applying the conventional random‐effects model to the resulting estimated log odds ratios. They further found that REML reduces, but does not remove, this bias (see their table II). Hence, our results for model 1 are qualitatively in agreement with these previous findings.

Also from Table 6, we can see that models 3, 4, 5, and 7 usually result in slight negative bias for τ ^2^. This makes intuitive sense because maximum likelihood estimates of unknown variance components are in general biased downwards. The settings where this is not the case are settings 7 (small studies), 8 (rare events), and 9 (very rare events). This makes sense because we can anticipate that the between‐study variance will be least precisely estimated when studies are small and/or events are rare. This means that the constraint that ${\hat{\tau }}^{2}$ must be positive has more potential to overcome the tendency for maximum likelihood estimates of variance components to be negatively biased. In setting 9, a comparison of the average ${\hat{\tau }}^{2}$ and the corresponding empirical standard errors indicates that these estimates are very skewed. This is especially the case for model 7, where a single very large ${\hat{\tau }}^{2}=23.46$ was obtained. Omitting this dataset substantially changes the 0.130 (0.780) entry in Table 6 to 0.106 (0.250), but this still indicates extreme skewness. Other models also provide very large estimates of the between‐study variance ( $5<{\hat{\tau }}^{2}<7$) for this very heterogeneous dataset and the defaults of the rma.glmm command result in a similar ${\hat{\tau }}^{2}=22.65$ for model 7. Hence, the estimate is genuine. The extreme skewness of estimates of τ ^2^ under models 3 to 7 in setting 9 has direct consequences for their notable positive bias (see Table 6). However, both the bias and skewness of estimates of τ ^2^ is substantially reduced in setting 9 when θ=log(2), where more events in the treatment group are observed (see Supporting Information). Furthermore, both the bias and skewness of estimates of τ ^2^ are also very much reduced in setting 8, relative to setting 9 (for both θ=0 and θ=log(2)), to the extent to which these are no longer a particular source of concern. We conclude that rare events result in difficulties for estimating τ ^2^ but also that the events must be very rare for this to be a serious cause for concern.

Model 6 is the only generalised linear mixed model that generally results in positive bias in the estimate of τ ^2^. This is notable because, as we have explained, in general maximum likelihood estimates of variance parameters are negatively biased. However, from (3), we can see that model 6 allows a fully unstructured covariance matrix, whereas in the bivariate representation of models 3 and 5, we can see these other models instead place constraints on this matrix. Model 3 places the strong constraint that the covariance is positive (σ ^2^). Model 5 allows this covariance to be negative but constrains this to be σ ^2^−τ ^2^/4, which is likely to be positive in the majority of settings in our simulation study where τ ^2^ is generally smaller than σ ^2^ (recall from Table 5 that the true σ ^2^=0.3^2^ in most settings). Hence, the constraints made by models 3 and 5 mean that their implied $\hat{\rho }$ in their bivariate representation (3) is either guaranteed or likely to be positive, which means that the final term in (4) for the estimated τ ^2^ will be negative. However, this constraint is not made by model 6, where $\hat{\rho }$ is likely to be imprecisely estimated and so well may be negative which would result in a larger ${\hat{\tau }}^{2}$. The unconstrained nature of the covariance matrix in model 6 therefore explains why it produces appreciably larger average estimates of τ ^2^ than models 3 and 5 and so provides slight positive bias, in many settings. Furthermore, model 6 allows a full bivariate model but in our simulation study, we produce data from reduced forms of this model, such as model 3, so that model 6 contains more variance component parameters than is necessary to describe the data. This may also contribute to the positive bias of estimates of τ ^2^ from model 6 in some settings.

#### 6.5.3 The bias of estimates of τ ^2^ from model 2 and a comparison with model 4

When the true value of τ ^2^=0.024, as is the case in all but settings 2, 3, and 15, average estimates of τ ^2^ that are less than around 0.01 were obtained from model 2, which constitutes considerable negative bias. An observation that is not revealed by Table 6 is that, in all but setting 15, it was a very rare event that the estimate of τ ^2^ from model 4 was found to be less than the corresponding estimate from model 2. Numerically, this appeared to occur more often than described below because occasionally, estimates from model 2 were reported that were essentially 0 and were apparently larger than estimates from model 4 that were reported as identically 0. Upon ignoring these cases, only 2 settings produced any datasets where the estimate of τ ^2^ from model 4 was less than that of model 2. This happened once in setting 9, for the very heterogeneous dataset that provided ${\hat{\tau }}^{2}=23.46$ for model 7 as described above. However, in setting 15 (extreme between‐study heterogeneity), this was commonplace [occurring 597/1000 times when θ=0 and 693/1000 times when θ=log(2)]. We conclude that unless the data are very heterogeneous, then it is highly unlikely that the estimate of τ ^2^ from model 2 is greater than that of model 4.

The Supporting Information gives full details of a mathematical explanation of why, in most settings, the estimate of τ ^2^ from model 2 is less than that of model 4. Briefly, we consider a very simple special case to obtain some analytical results, where we assume that all treatment and control arms are of similar size and that this size is large enough to use normal approximations for all empirical log odds in all arms. This means that we can use the same within‐study arm variance s ^2^ throughout our analysis. Under these assumptions we find, in meta‐analyses with a reasonably large number of studies, that the maximum likelihood estimate of τ ^2^ under model 4 is approximately the DerSimonian and Laird estimator under model 1. Hence, the properties of estimators under model 4 will be acceptable. However, we find that there is downward bias in the estimate of τ ^2^ under model 2, even as the number of studies k tends towards infinity. We also find that the estimate of τ ^2^ will be approximately s ^2^ larger when fitting model 4 compared to model 2. In our simulation study, this difference is around 1/(275×0.2×0.8)≈0.02 which is nicely in agreement with the downward bias in ${\hat{\tau }}^{2}$ that we observe in Table 6 in the majority of settings. However, this difference is negligible in relation to the true τ ^2^=2 in setting 15 and our theory essentially predicts no difference between these 2 estimates in this setting. The assumptions made in our mathematical analysis (all study arms of similar size, normal approximations for all empirical log odds) become less valid for very large τ ^2^, and in fact the average ${\hat{\tau }}^{2}$ from model 2 is slightly greater than this average from model 4 in setting 15 (Table 6). Despite this, our mathematical analysis nicely supports the surprising finding that the estimate of τ ^2^ is negatively biased by around 0.02 in the majority of settings.

In Section 4, we warned the reader that models 2 and 4 do not satisfy the regularity conditions for maximum likelihood estimation to have its usual good properties. Our unsettling finding about the bias of the estimate of τ ^2^ under model 2 in most simulation settings would seem to be a consequence of this. Our simulation study is sufficient to identify that, when fixed effects are assumed for the γ_i_, apparently innocuous assumptions about the form of the random effect can have serious consequences for the estimation. On the basis of this simulation study, it seems that model 4 performs much better than model 2.

#### 6.5.4 Precision of estimates of θ and τ ^2^

The empirical standard errors of the estimates of *θ* in Table 6 are generally similar across all models. This is as expected because the precision of estimates depends primarily on the data available, rather than the specific model used, but recall that a concern was that model 7 might result in a slight loss of information by conditioning on a statistic that is only approximately ancillary. This loss of information would appear to be negligible, as expected because very little information about the odds ratio is contained in the total number of events in 2‐by‐2 tables. In setting 9 (very rare events), the empirical standard errors of $\hat{\theta }$ are appreciably larger in models 2 to 7 than in model 1, but we will see below that nominal 95% confidence intervals from model 1 are conservative in this setting. This observation would seem to be another consequence of the normal approximations used in model 1 describing the data poorly in this setting.

The conclusions for the empirical standard errors of the estimates of *τ* ^2^ are a little more complicated. In most settings, model 2 provides smaller empirical standard errors for ${\hat{\tau }}^{2}$ but the negative bias in the point estimates under this model render this observation moot. Models 3 to 7 generally provide very similar empirical standard errors that are smaller than those from model 1, which suggests that modelling the binomial data using generalised linear mixed models has the potential to provide more precise estimates of *τ* ^2^. However, this observation is offset by the negative bias of ${\hat{\tau }}^{2}$ under these models. The considerable positive bias of estimates of *τ* ^2^ from models 3 to 7, that is largely due to their skewness, in setting 9 (very rare events) raises some concerns about using these models in this situation.

**Figure 1:**
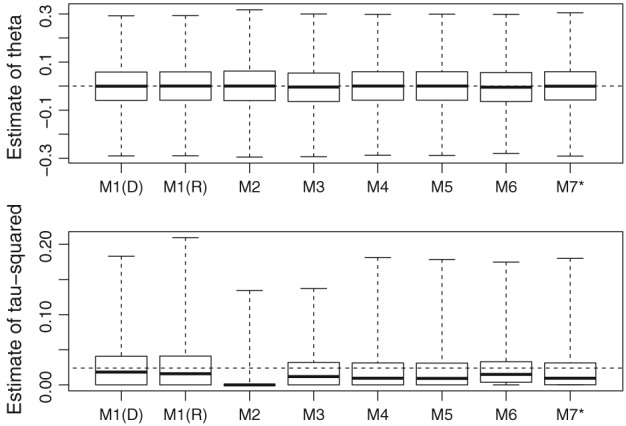
Box plots of the estimates of θ (top) and τ ^2^ (bottom) from setting 1 (the defaults). The true values of θ=0 and τ ^2^=0.024 are shown as dashed lines. M1(D) and M1(R) indicate that model 1 has been fitted using the DerSimonian and Laird and the REML estimator, respectively. M7* indicates that model 7 has been supplemented with results from the Peto approximation

To illustrate the bias and precision of estimates visually, box plots of the estimators from all models in setting 1 (the defaults) and *θ*=0 are shown in Figure 1, for *θ*(top) and *τ* ^2^ (bottom). The true values of *θ*=0 and *τ* ^2^=0.024 are shown as dashed lines. Figure 1 confirms that there is little to choose between the models in terms of the point estimate of *θ* but also indicates that the case for the estimate of *τ* ^2^ is more complicated. Figure 1 captures the skewness in the point estimates of *τ* ^2^. Figure 1 also illustrates the extreme negative bias of ${\hat{\tau }}^{2}$ under model 2 in this setting.

### 6.6 Coverage probability of nominal 95% confidence intervals for θ

The coverage probabilities of nominal 95% confidence intervals are shown in Table 7. All models involve some form of approximation when computing these confidence intervals; for example, models 2 to 7 use the asymptotic theory of maximum likelihood, and so some departure from the nominal coverage probability was anticipated. With the exception of model 2, all methods perform similarly in settings other than the challenging settings 9 (very rare events) and 15 (extreme between‐study heterogeneity). It is perhaps disappointing that the generalised linear mixed models have not resulted in a more tangible improvement in the majority of settings. As expected, confidence intervals are generally slightly conservative in setting 2 where *τ* ^2^=0 and perform least well in settings 3 and 15 where *τ* ^2^ is large and extremely large, respectively, but otherwise models 1 and models 3 to 7 generally fail to achieve the nominal coverage probability by around 1% or 2%, as might be expected. In setting 4, where there are just *k*=3 studies, the coverage probabilities generally appear to be a little lower than this, but given the very small number of studies, the models have performed remarkably well. An interesting observation is that nominal 95% confidence intervals from model 1 are conservative in setting 9. This is consistent with the findings of Bradburn et al^40^ (their figure 3) and is further evidence that normal approximations perform poorly in this setting. Models 2 to 7 retain the nominal coverage probability much more accurately in setting 9, which indicates that the use of generalised linear mixed models may be especially advantageous in situations where the events are rare. This is as might be anticipated, but this finding is somewhat offset by the conclusion that the accurate estimation of *τ* ^2^ is especially challenging in this type of situation. In setting 15, models 2 to 7 fail to achieve their nominal coverage probability by around 5%. Model 1 (REML) appears to fare slightly better in this setting but the DerSimonian and Laird method performs worst, which is clearly a consequence of the notable downward bias of the corresponding estimator of *τ* ^2^ in setting 15.

**Table 7:** Simulation study results. Actual coverage probability of 95% confidence intervals for θ. The average model based standard errors, as a percentage of the corresponding empirical standard errors, are shown in parentheses. Model 7^∗^ indicates that inferences for model 7 have been supplemented with results from the ‘Peto approximation’ described in section 3.7.3, as explained in Section 6.3

	Model 1	Model 1	Model 2	Model 3	Model 4	Model 5	Model 6	Model 7^∗^
Setting	(D & L)	(REML)	(ML)	(ML)	(ML)	(ML)	(ML)	(ML)
1	0.933 (98)	0.931 (98)	0.881 (81)	0.923 (93)	0.925 (94)	0.925 (93)	0.926 (97)	0.920 (94)
2	0.957 (105)	0.957 (104)	0.945 (97)	0.952 (102)	0.953 (102)	0.953 (102)	0.956 (105)	0.953 (103)
3	0.910 (94)	0.913 (95)	0.839 (80)	0.899 (90)	0.892 (90)	0.895 (90)	0.894 (90)	0.895 (91)
4	0.935 (106)	0.937 (106)	0.907 (88)	0.921 (95)	0.921 (95)	0.922 (95)	0.947 (103)	0.922 (95)
5	0.939 (104)	0.937 (104)	0.913 (89)	0.933 (99)	0.932 (97)	0.932 (97)	0.949 (103)	0.929 (98)
6	0.943 (102)	0.947 (102)	0.894 (84)	0.936 (100)	0.943 (99)	0.943 (99)	0.942 (100)	0.941 (103)
7	0.955 (105)	0.957 (105)	0.930 (92)	0.944 (98)	0.943 (100)	0.942 (100)	0.956 (104)	0.944 (100)
8	0.952 (102)	0.950 (102)	0.911 (89)	0.937 (96)	0.937 (97)	0.936 (96)	0.958 (102)	0.937 (97)
9	0.986 (121)	0.986 (121)	0.948 (95)	0.958 (99)	0.955 (102)	0.957 (103)	0.968 (102)	0.961 (101)
10	0.939 (101)	0.944 (100)	0.896 (85)	0.934 (97)	0.923 (94)	0.936 (97)	0.952 (99)	0.935 (97)
11	0.936 (101)	0.933 (101)	0.898 (84)	0.930 (97)	0.923 (96)	0.927 (98)	0.939 (100)	0.925 (98)
12	0.931 (101)	0.933 (101)	0.881 (83)	0.927 (97)	0.922 (95)	0.923 (97)	0.931 (98)	0.926 (97)
13	0.946 (101)	0.943 (101)	0.906 (85)	0.941 (97)	0.935 (97)	0.936 (97)	0.945 (99)	0.936 (98)
14	0.920 (97)	0.917 (96)	0.880 (80)	0.906 (89)	0.911 (92)	0.911 (92)	0.916 (93)	0.906 (92)
15	0.876 (84)	0.915 (98)	0.899 (92)	0.898 (93)	0.900 (92)	0.900 (92)	0.899 (92)	0.901 (94)

Abbreviation: REML, restricted maximum likelihood.

Model 2 has resulted in notable under coverage in all settings. This can be explained by the large negative bias in the estimate of *τ* ^2^ in settings 1 to 14, and because all methods provide under coverage in setting 15. Although the coverage probability for model 2 does not drop below around 84%, the general picture is that this model has performed poorly. Another observation is that there appears to be some tendency for model 6 to provide slightly larger coverage probabilities that are closer to the nominal level. This model would appear to benefit from its upward bias of the estimate of *τ* ^2^ that we discussed in the previous subsection.

The average model‐based (as reported when fitting models) standard errors, as a percentage of the corresponding empirical standard errors, are shown in parentheses in Table 7. In general, these are close to 100%, which further indicates that the inference from our models is accurate. However, percentages in the range 80% to 90% are common from model 2, which reinforces the conclusion that this model has performed poorly. Furthermore, this ratio is 121% for model 1 in setting 9, which is consistent with the overcoverage of nominal 95% confidence intervals for model 1 in this setting.

### 6.7 Robustness to the inclusion or exclusion of double‐zero studies in setting 9

As explained above, in setting 9 a non‐trivial number of simulated datasets contained no events. As also explained above, by following our defaults, we included these studies when fitting models 1, 3, and 6 but it is probably better to exclude them. We therefore refitted these 3 models in setting 9 where all double‐zero studies were excluded. We found that the differences between the results that exclude (results not shown) and include them (Tables 6 and 7 and Supporting Information), although perceptible, were not substantial and do not alter any of the conclusions. For example, we noted above that model 1 resulted in a bias towards the null of 0.06 to 2 decimal places when θ=log(2). It was suspected that some of this bias towards the null may be due to including double‐zero studies. This bias noticeably fell but was still 0.06 (to 2 decimal places) upon excluding these studies. This small reduction can be explained by the fact that double‐zero studies are unusual when θ=log(2) in setting 9, and these studies carry relatively little weight in many analyses where ${\hat{\tau }}^{2}$ is small or 0.

### 6.8 Conclusions from the simulation study

The finding that models 2 and 4 perform so differently raises serious concerns about modelling study specific event rates using fixed effects. This is because apparently innocuous changes to the model result in substantial differences in the estimation of *τ* ^2^. Our simulation study suggests that model 4 performs substantially better than model 2, so that until more evidence becomes available, we suggest that model 4 should be preferred in application.

The simulation study also suggests that the recovery of intertrial information can be a potential source of bias in models that instead use random effects to describe study specific event rates, but we also conclude that this bias is likely to be small. In practical applications, model 6 may be preferred to models 3 and 5 on the grounds that it does not place unnecessary constraints on the covariance structure in bivariate random‐effects models of this type, but this appears to come at the price of positive bias in the estimate of *τ* ^2^ in most settings. However, this upward bias, although appreciable, is not very large and some may consider this to be a desirable consequence because this appears to result in slightly higher confidence interval coverage probabilities for *θ* that are closer to the nominal level. Model 7 performs well and the information lost by the conditioning on a statistic that is not quite ancillary appears to be negligible. However, the computation required to fit this model can be fragile so that some statistical expertise is needed to assess this.

It is therefore not possible to recommend a single model on the grounds that it is uniformly better than others. However, since model 2 has been found to perform poorly, and model 6 appears to be a more conservative version of models 3 and 5 that places no unnecessary constraints on the covariance structure, it would seem reasonable to suggest that models 4 and 6, and also model 7 when statistical expertise is available, are all fully viable alternatives to the conventional random‐effects model (model 1). Since it is evident that the modelling assumptions made in random‐effects meta‐analyses can have implications for the conclusions, the most prudent approach would seem to be to fit several of the random‐effects models that we have proposed and assess the implications in the context of a sensitivity analysis. However, the conventional model (model 1) appears to retain its usefulness because it is so simple to implement and has not been markedly improved upon by the other models unless the event is very rare or, in the case of the DerSimonian and Laird method, if the data are extremely heterogeneous. This is likely to be a disappointing finding for those who might advocate the wholesale abandonment of the conventional random‐effects model and the adoption of more sophisticated models such as those we present here.

## 7 DISCUSSION

We have investigated the use of 7 random‐effects models for meta‐analysis that estimate the summary odds ratio. We have found, through simulation and analytical investigations, that one of these models (model 2) performs poorly. Our investigation must leave us feeling a little uncomfortable when using generalised linear mixed models for meta‐analysis that include fixed‐study effects. This is because we have seen that apparently innocuous changes in the model form can have serious consequences for maximum likelihood estimation.

An alternative is to use random‐study effects, but we anticipate that some readers will object these to on the grounds that intertrial information is then recovered. To some, it may be considered undesirable, or even nonaxiomatic, to allow the use of intertrial information regardless of whether or not bias is invoked. We anticipate that discussion relating to this issue will continue, where the arguments for and against random‐study effects are likely to follow along the same lines as in network meta‐analysis.(41, 42) If random study effects are to be used then model 6 is likely to be considered preferable to models 3 and 5, on the grounds that it is a perfectly applicable generalisation of these models that avoids making unnecessary independence assumptions; likelihood ratio tests and Akaike information criterion statistics could be used for determining model choice in situations where model reduction is considered desirable. Model 7 would seem to be a desirable alternative but the numerical challenges involved in fitting this model make it difficult to recommend for routine use. If a sensitivity analysis is to be performed using a variety of statistical models, then we suggest that models 1, 4, 6, and 7 are more than adequate for this purpose. The estimation of *τ* ^2^ in particular can be sensitive to the model choice, and we advocate sensitivity analysis to explore this.

Our conclusion that model 2 performs poorly, and the consequent concerns about using fixed‐study effects, has considerable implications for individual patient data (IPD) meta‐analyses.^43^ Model 2 may be extended to include study‐level covariates, and then used in a wide variety of applications of this type. Our work therefore opens up IPD meta‐analyses that have used models based on model 2 to unexpected criticism. It will be of particular interest to see how the IPD meta‐analysis community responds to our discoveries.

We have focussed on binary outcome data where the summary odds ratio is of interest. Further work should focus on alternative measures of association, such as the relative risk, and more importantly on other forms of outcome data. For example, generalised linear mixed models can be used for count data^26^ and linear mixed models can be used for continuous data. When the outcome data are continuous, REML^44^ is generally preferred to maximum likelihood estimation on the grounds that it helps to avoid the downward bias of estimated variance components. Although continuous data are not the focus of this paper, an obvious question is whether REML would resolve the issues with models for continuous outcomes that are analogous to model 2. In the Supporting Information, we extend our mathematical analysis to incorporate REML estimation, where we find that REML does resolve these difficulties. This conclusion was confirmed in a small‐scale simulation study using continuous data, that was intended to emulate the first simulation study setting. Here, a model for continuous data, akin to model 2, was fitted to simulated IPD using the *nlme* package, using syntax of the form 



lme(Y ~ factor(study)+factor(treat), random = ~ treat-1|study),





where options method=“ML” and method=“REML” can be used to specify that maximum likelihood or restricted maximum likelihood is to be used, respectively. Models akin to model 4 can be fitted by instead specifying the option random= treat12‐1|study. Briefly, we found notable downward bias in estimates of *τ* ^2^ when fitting models akin to model 2 when using maximum likelihood estimation, in a similar way, as for binary outcome data in the simulation study. However, when using models akin to model 4 using maximum likelihood, or REML in conjunction with a model akin to either model 2 or 4, there was no serious bias in this estimation. Further work that examines continuous outcome data is highly desirable, but our analysis and small‐scale simulation study indicates that the use of REML entirely avoids any serious issues relating to bias when using fixed‐study effects and the outcome data are continuous. The examination of other types of likelihood function is another interesting avenue for further work. In particular, alternative forms of estimation of model 2 that, like REML, take into account the degrees of freedom lost when including many fixed effects in the model have the potential to overcome the problems that we have encountered. We leave this as an important avenue for further work. Another possibility to explore is the implications of the methods used to integrate out the random effects; variations of the methods used for this will have some implications for the accuracy of the results. Further work could examine the tradeoffs between using methods that are accurate and methods that are computationally less demanding.

In conclusion, more sophisticated models for meta‐analysis, such as those that we have investigated, should be considered for use in application more often than is currently the case. In addition to highlighting some concerns about the use of generalised linear mixed models in meta‐analysis, we also hope that this paper will serve to further draw them to the attention of applied analysts. These models have the potential to overcome some of the limitations of the current standard approach, but they also have their disadvantages. We provide R, Stata, and SAS codes in the Supporting Information to facilitate the use of generalised linear mixed models in applied meta‐analyses.

## Supporting information



Table 1. Simulation study results. The top half of the table shows the mean estimate of the average log‐odds ratio θ minus log(2), that is the bias of the estimate of θ; Monte Carlo standard errors are shown in parentheses. The bottom half of the table shows the mean estimate of τ^2^. The true value is θ=log(2) ≈0.693; results for θ=0 are shown in the main paper. Model 7^*^ indicates that inferences for model 7 have been supplemented with results from the 'Peto approximation'.
Table 2. Simulation study results. Actual coverage probability of 95% confidence intervals. The average model based standard errors, as a percentage of the corresponding empirical standard errors, are shown in parentheses. Model 7^*^ indicates that inferences for model 7 have been supplemented with results from the 'Peto approximation'
