# Supplementary material for: A comparison of seven random‐effects models for meta‐analyses that estimate the summary odds ratio
Source: Stat Med. 2018 Jan 8;37(7):1059–85. doi: 10.1002/sim.7588 (PMC5841569; doi:10.1002/sim.7588)
Supplement: Supplementary file 1 — Table 1. Simulation study results. The top half of the table shows the mean estimate of the average log‐odds ratio θ minus log(2), that is the bias of the estimate of θ; Monte Carlo standard errors are shown in parentheses. The bottom half of the table shows the mean estimate of τ2. The true value is θ=log(2) ≈0.693; results for θ=0 are shown in the main paper. Model 7* indicates that inferences for model 7 have been supplemented with results from the 'Peto approximation'. Table 2. Simulation study results. Actual coverage probability of 95% confidence intervals. The average model based standard errors, as a percentage of the corresponding empirical standard errors, are shown in parentheses. Model 7* indicates that inferences for model 7 have been supplemented with results from the 'Peto approximation' [file SIM-37-1059-s001.zip › Analytical supplement4.pdf]

Statistics in Medicine

Research Article

Received XXXX

(www.interscience.wiley.com) DOI: 10.1002/sim.0000

Supplementary materials: Analytical investigation into models two and four

In this appendix we explain why the estimates of τ^2 from models two and four differ so substantially, and also why the estimate of τ^2 from model two is biased downwards.

We let y_{ij} denote the estimated log odds of an event in the ith study and the j treatment group, where $j = 0, 1$ continues to represent the control and treatment groups. In order to obtain analytical results, we assume that the treatment groups are large enough to use normal approximations for the y_{ij} and we further simplify matters by assuming that all study groups in all studies are of similar size, so that we can use the same within study arm variance s^2 for all y_{ij}. We can therefore approximate models two and four using normal approximations and so assume the modelling framework

$$y_{ij} = \gamma_i + j\theta + (j - a)\epsilon_i + e_{ij} \tag{1}$$

where $\epsilon_i \sim N(0, \tau^2)$ and $e_{ij} \sim N(0, s^2)$; $a = 0$ is then model two, $a = 0.5$ is model four and $-1 \leq a \leq 1$ describes a family of other closely related models. Writing $\mathbf{y}_i = (y_{i0}, y_{i1})^t$, we therefore have that $\mathbf{y}_i$ is approximately normally distributed with $\mathrm{E}[\mathbf{y}_i] = (\gamma_i, \gamma_i + \theta)^t$ and

$$\mathbf{V} = \begin{bmatrix} a^2\tau^2 + s^2 & -a(1-a)\tau^2 \\ -a(1-a)\tau^2 & (1-a)^2\tau^2 + s^2 \end{bmatrix} \tag{2}$$

where $\mathbf{V}$ is the covariance matrix for all studies. We will derive the profile likelihood for τ^2, also as a function of a, in order to determine the implications of a for the estimation of τ^2. In particular, it will be of interest to compare the cases $a = 0$ and $a = 0.5$.

We first derive the estimates $\hat{\gamma}_i$ for fixed values of θ and τ^2, in order to later obtain the profile likelihood for θ and τ^2. For convenience we define $z_{i1} = y_{i1} - \theta$. Then

$$\begin{bmatrix} y_{i0} \\ z_{i1} \end{bmatrix} \sim N(\mathbf{1}\gamma_i, \mathbf{V}) \tag{3}$$

where $\mathbf{1} = (1, 1)^t$. For fixed values of θ and τ^2, only the ith study provides information for, and so contributes to, the estimate $\hat{\gamma}_i$. It is a standard result that with design matrix $\mathbf{X}$, and outcome data $\mathbf{Y}$ with variance $\mathbf{V}$, the estimate of the vector of regression parameters is $(\mathbf{X}^t\mathbf{V}^{-1}\mathbf{X})^{-1}\mathbf{X}^t\mathbf{V}^{-1}\mathbf{Y}$. In model (3) the design matrix is $\mathbf{1}$, so from this model and the standard result for a weighted regression, after a little algebra we can derive

$$\hat{\gamma}_i(\tau^2, \theta) = c(\tau^2)z_{i1} + (1 - c(\tau^2))y_{i0}$$

where $c(\tau^2) = (a\tau^2 + s^2)/(\tau^2 + 2s^2)$; $\hat{\gamma}_i(\tau^2, \theta)$ depends on τ^2 via $c(\tau^2)$ and on θ via $z_{i1} = y_{i1} - \theta$. For fixed values of θ and τ^2, the vector of residuals for the ith study are therefore given by

$$\mathbf{r}_i(\theta, \tau^2) = \begin{bmatrix} y_{i0} \\ y_{i1} \end{bmatrix} - \begin{bmatrix} \hat{\gamma}_i(\tau^2, \theta) \\ \hat{\gamma}_i(\tau^2, \theta) + \theta \end{bmatrix} = \delta_i \begin{bmatrix} -c(\tau^2) \\ 1 - c(\tau^2) \end{bmatrix}$$

where $\delta_i = y_{i1} - \theta - y_{i0}$. The contribution of the ith study to minus two times the profile log-likelihood for θ and τ^2, $\ell_i(\theta, \tau^2)$, is therefore

$$-2\ell_i(\theta, \tau^2) = \log(|\mathbf{V}|) + \mathbf{r}_i^t(\theta, \tau^2)\mathbf{V}^{-1}\mathbf{r}_i(\theta, \tau^2) \tag{4}$$

where the γ_i in the log-likelihood have been replaced by $\hat{\gamma}_i(\theta, \tau^2)$.

We can easily evaluate $\log(|\mathbf{V}|) = \log(b\tau^2 + s^2) + \log(s^2)$, where $b = a^2 + (1 - a)^2$. However the second term in this profile log-likelihood, $\mathbf{r}_i^t(\theta, \tau^2)\mathbf{V}^{-1}\mathbf{r}_i(\theta, \tau^2)$ is harder to calculate. One way to evaluate this is to write $\mathbf{V}^{-1}$ in terms of its spectral decomposition

$$\mathbf{V}^{-1} = \lambda_1\mathbf{e}_1\mathbf{e}_1^t + \lambda_2\mathbf{e}_2\mathbf{e}_2^t$$

where λ_1 and λ_2 are the eigenvalues of $\mathbf{V}^{-1}$ and $\mathbf{e}_1$ and $\mathbf{e}_2$ are the corresponding normalised eigenvectors. This way of writing $\mathbf{V}^{-1}$ is convenient because its eigenvectors and normalised eigenvectors have very simple expressions, $\lambda_1 = 1/s^2$, $\lambda_2 = 1/(s^2 + b\tau^2)$,

Copyright © 0000 John Wiley & Sons, Ltd.

$\mathbf{e}_1 = (a(1-a), a^2)^t/(a\sqrt{b})$ and $\mathbf{e}_2 = (-a^2, a(1-a))^t/(a\sqrt{b})$. We can then write the second term in the profile log-likelihood as

$$\mathbf{r}_i^t(\theta,\tau^2)\mathbf{V}^{-1}\mathbf{r}_i(\theta,\tau^2) = \mathbf{r}_i^t(\theta,\tau^2)(\lambda_1\mathbf{e}_1\mathbf{e}_1^t + \lambda_2\mathbf{e}_2\mathbf{e}_2^t)\mathbf{r}_i(\theta,\tau^2) = \lambda_1(\mathbf{e}_1^t\mathbf{r}_i(\theta,\tau^2))^2 + \lambda_2(\mathbf{e}_2^t\mathbf{r}_i(\theta,\tau^2))^2$$

After some tedious algebra this can be written as

$$\mathbf{r}_i^t(\theta,\tau^2)\mathbf{V}^{-1}\mathbf{r}_i(\theta,\tau^2) = \delta_i^2\left(\frac{(a(a-c(\tau^2)))^2}{s^2a^2b} + \frac{(a(2ac(\tau^2)-c(\tau^2)-a+1))^2}{(s^2+b\tau^2)a^2b}\right)$$

Then, using the definition of $c(\tau^2)$ we can write

$$\mathbf{r}_i^t(\theta,\tau^2)\mathbf{V}^{-1}\mathbf{r}_i(\theta,\tau^2) = \delta_i^2\left(\frac{s^2(1-2a)^2}{b(\tau^2+2s^2)^2} + \frac{(2a\tau^2(1-a)-\tau^2-s^2)^2}{b(s^2+b\tau^2)(\tau^2+2s^2)^2}\right) \tag{5}$$

Next we use the identity

$$b(\tau^2+2s^2)(s^2+b\tau^2) = s^2(1-2a)^2(s^2+b\tau^2) + (2a\tau^2(1-a)-\tau^2-s^2)^2$$

This identity can be checked by expanding both sides, recalling that $b = a^2 + (1-a)^2$, and confirming that the coefficients in the resulting quadratics in τ^2 in both sides are identical. Upon making use of this identity we can drastically simplify (5) which becomes

$$\mathbf{r}_i^t(\theta,\tau^2)\mathbf{V}^{-1}\mathbf{r}_i(\theta,\tau^2) = \delta_i^2/(\tau^2+2s^2) \tag{6}$$

From (1) and (2) we have $\mathrm{Var}(y_{i1}-\theta-y_{i0}) = \mathrm{Var}(\delta_i) = \tau^2+2s^2$, so that the simplification in (6) is natural. This means that (4) is simply

$$-2\ell_i(\theta,\tau^2) = \log(b\tau^2+s^2) + \log(s^2) + \delta_i^2/(\tau^2+2s^2)$$

Hence the profile log-likelihood of the data from all k studies is

$$-2\ell(\theta,\tau^2) = -2\sum_{i=1}^{k}\ell_i(\theta,\tau^2) = k\log(b\tau^2+s^2) + k\log(s^2) + \sum_{i=1}^{k}\delta_i^2/(\tau^2+2s^2) \tag{7}$$

Differentiating with respect to θ, for a fixed value of τ^2 we obtain $\hat{\theta} = \bar{y}_1 - \bar{y}_0$, where $\bar{y}_o = \sum_i y_{io}/k$; in fact we obtain this estimate irrespective of the value of τ^2. Replacing $\hat{\theta}$ with its estimate for the fixed value of τ^2, we obtain the profile log-likelihood for τ^2 of

$$-2\ell(\tau^2) = k\log(b\tau^2+s^2) + k\log(s^2) + \mathrm{TSS}/(\tau^2+2s^2) \tag{8}$$

where TSS is the (unweighted) total sum of squares of the study specific treatment effects $y_i = y_{i1} - y_{i0}$, that is $\mathrm{TSS} = \sum(y_i-\bar{y})^2$, where $\bar{y}$ is the mean of the y_i. Differentiating (8) with respect to τ^2, we derive the estimating equation

$$f(\hat{\tau}^2; b) = \frac{k}{\hat{\tau}^2+s^2/b} - \frac{\mathrm{TSS}}{(\hat{\tau}^2+2s^2)^2} = 0 \tag{9}$$

We have $s^2 > 0$ so it is clear that $f(\hat{\tau}^2; b)$ is increasing in b. Equation (9) results in a quadratic in $\hat{\tau}^2$. Assuming k is large and writing $s_y^2 = \mathrm{TSS}/(k-1) \approx \mathrm{TSS}/k$ as the sample variance of the y_i, solving this quadratic gives

$$\hat{\tau}^2 = \frac{s_y^2}{2} - 2s^2 + \frac{1}{2}s_y\sqrt{s_y^2 + 4s^2(1/b-2)} \tag{10}$$

where the case $b = 1/2$ confirms that we have avoided the superfluous solution of the quadratic equation which is instead $\hat{\tau}^2 = -2s^2$, which is negative and therefore clearly spurious. The solution to (10) would be truncated to zero if negative, and from this equation is it obvious that the 'untruncated' estimate of τ^2 is decreasing in b; hence to obtain a large $\hat{\tau}^2$ we require a small value of b. Recalling again that $b = a^2 + (1-a)^2$ and $-1 \le a \le 1$, the minimum value of b is $b = 1/2$ which is achieved when $a = 1/2$, which corresponds to model four. Hence the maximum estimate of τ^2, over a, for any dataset is obtained when assuming model four. Although we use normal approximations and assume that all treatment groups in all studies are of similar size, this explains why it is a very rare event that model two results in a larger estimate of τ^2 than model four in our simulation study in most settings: For the special case we have considered here, upon using normal approximations, it guaranteed that model four will result in a larger estimate of τ^2 than model two. However in setting 15 the difference between these two estimates predicted by our analysis is negligible (see below) and our simplifying assumptions are not accurate in this final setting. As explained in the main paper, in setting 15 the estimate of τ^2 from model four is frequently less than the one from model two.

In order to further understand the implications of the value of a, we continue a little further. In a standard DerSimonian and Laird meta-analysis, the outcome data are the y_i and the within-study variances are all $2s^2$ in this simple setting. Furthermore, the Q statistic that is used to estimate τ^2 is closely related to TSS when all variances are the same and the DerSimonian and Laird estimator drastically simplifies. Specifically, the DerSimonian and Laird estimator is equal to the sample variance of the outcome

Copyright © 0000 John Wiley & Sons, Ltd.

data minus the (common to all studies) within-study variance. Hence continuing to assume that the sample size is large so that $k-1 \approx k$, we can approximate the untruncated DerSimonian and Laird estimator as $\hat{\tau}^2_{DL} = s_y^2 - 2s^2 \approx \text{TSS}/k - 2s^2$. Replacing TSS with $k(\hat{\tau}^2_{DL} + 2s^2)$ in (9) immediately yields the result that the DerSimonian and Laird estimator of τ^2 is effectively obtained when $b = 1/2$. Hence if $b = 1/2$ and the sample size is large, the estimate of τ^2 is approximately unbiased. For any other value of b the estimate of τ^2 is negatively biased, even asymptotically. $b = 1/2$ corresponds to model four, which suggests that this model should be preferred to model two, exactly as we found in the simulation study.

Finally note that, for the special case considered, (9) depends on a only through b. This suggests that, more generally, it is likely to be the value of $b = a^2 + (1-a)^2$ that is important. Writing $c = 1/b$, equation (10) gives the very simple derivative

$$\left.\frac{d\hat{\tau}^2}{dc}\right|_{c=2} = s^2$$

Noting that $a = 1/2$ (so that $b = 1/2$ or $c = 2$) provides model four, and $a = 1$ (so that $b = 1$ or $c = 1$) provides model two, a linear approximation at $c = 2$ (and ignoring the impact of truncating estimates of τ^2 to zero that would otherwise be negative) means that we anticipate the estimate of τ^2 will be s^2 (the variance of the log-odds in either arm) larger when fitting model four ($c = 2$) compared to model two ($c = 1$). In our simulation study this difference is around $1/(275 \times 0.2 \times 0.8) \approx 0.02$, which is nicely in agreement with what we observe in most simulation settings. In setting 15 this difference is negligible in relation to the true $\tau^2 = 2$.

REML estimation

When the outcome data are modelled as continuous, REML can be used instead of maximum likelihood. As explained by Jennrich and Schluchter [1], REML introduces an additional term to (minus two times) the log-likelihood, which given the common variance assumption made here is given by

$$\log|\sum_{i=1}^{k} \mathbf{X}_i^t \mathbf{V}^{-1} \mathbf{V}_i| \tag{11}$$

where $\mathbf{X}_i$ is the design matrix for study i. Although we have profiled out the γ_i and θ to produce the profile log-likelihood (8), the restricted likelihood is based on the likelihood of the residuals for a given value of τ^2, and so we also take γ_i and θ to be their estimated values given τ^2 when computing the restricted likelihood. Hence the profile log-likelihood that we have derived is two of the terms of the corresponding restricted log-likelihood function (given in a more general form on page 812 of Jennrich and Schluchter [1]) and we add (11) to (8) to obtain the restricted log-likelihood function required.

By defining the vector of regression coefficients as $(\gamma_1, \gamma_2, \cdots, \gamma_k, \theta)^t$, this means that $\mathbf{X}_i$ is a $2 \times (k+1)$ matrix where the entries in the ith column are both 1, the entry in the second row and $(k+1)$th column is also 1 and all other entries are 0. We can then evaluate $\sum_{i=1}^{k} \mathbf{X}_i^t \mathbf{V}^{-1} \mathbf{X}_i$ as a $(k+1) \times (k+1)$ matrix where all entries that are *not* along the main diagonal, or in the final row or column, are all zero. Furthermore, all the entries along the main diagonal, except the final one, are $(\tau^2 + 2s^2)/(s^2(s^2 + b\tau^2))$, the final entry along the main diagonal is $(k(a^2\tau^2 + s^2))/(s^2(s^2 + b\tau^2))$, and all other entries in the final row and column are $(a\tau^2 + s^2)/(s^2(s^2 + b\tau^2))$.

We can then evaluate the additional term introduced by REML as $\log|\sum_{i=1}^{k} \mathbf{X}_i^t \mathbf{V}^{-1} \mathbf{X}_i|$. One way to evaluate the determinant of $\sum_{i=1}^{k} \mathbf{X}_i^t \mathbf{V}^{-1} \mathbf{X}_i$ is to use elementary row operations (which do not change the determinant) to obtain zeroes for all entries along the final row (or column) except the last entry. Then by cofactor expansion, the determinant is just the product of the main diagonal entries. The algebra required is tedious and we give some details here. We can simplify matters using the result that $|c\mathbf{M}| = c^d|\mathbf{M}|$ where d is the dimension of $\mathbf{M}$. Hence the determinant of $\sum_{i=1}^{k} \mathbf{X}_i^t \mathbf{V}^{-1} \mathbf{X}_i$ is equal to the product of $(1/(s^2(s^2 + b\tau^2)))^{(k+1)}$ and $|\mathbf{M}|$, where all entries of $\mathbf{M}$ that are *not* along the main diagonal, or in the final row or column, are all zero. Furthermore, all the entries along the main diagonal of $\mathbf{M}$, except the final one, are $\alpha_a = \tau^2 + 2s^2$, the final entry along the main diagonal is $\alpha_b = k(a^2\tau^2 + s^2)$ and all other entries in the final row and column are $\alpha_c = a\tau^2 + s^2$. Using the type of cofactor expansion described above, we can then evaluate $|\mathbf{M}| = \alpha_a^{k-1}(\alpha_a\alpha_b - k\alpha_c^2)$. After a little more algebra, and making use of the identity $\alpha_a\alpha_b - k\alpha_c^2 = k(s^2(s^2 + b\tau^2))$, we obtain

$$\log|\sum_{i=1}^{k} \mathbf{X}_i^t \mathbf{V}^{-1} \mathbf{V}_i| = -k\log(s^2) - k\log(b\tau^2 + s^2) + \log(k) + (k-1)\log(\tau^2 + 2s^2) \tag{12}$$

Then adding the extra term in (12) to the profile log-likelihood (8), and ignoring all terms that do not involve τ^2, gives

$$-2\ell(\tau^2) = (k-1)\log(\tau^2 + 2s^2) + \text{TSS}/(\tau^2 + 2s^2) \tag{13}$$

Note now the restricted log-likelihood (13) does not depend on b. Representing $\hat{\tau}^2_{DL} = s_y^2 - 2s^2 = \text{TSS}/(k-1) - 2s^2$ and following similar arguments as above means that we effectively obtain the DerSimonian and Laird estimator for all b without

Copyright © 0000 John Wiley & Sons, Ltd.

assuming large samples. This indicates that REML resolves the difficulties when using model two in situations where it is applicable.

References

1. Jennrich RI, Schluchter MD. Unbalanced repeated-measures models with structured covariance matrices. *Biometrics* 1986; 42: 805–820.

 Copyright © 0000 John Wiley & Sons, Ltd.
