# Supplementary material for: A comparison of seven random‐effects models for meta‐analyses that estimate the summary odds ratio
Source: Stat Med. 2018 Jan 8;37(7):1059–85. doi: 10.1002/sim.7588 (PMC5841569; doi:10.1002/sim.7588)
Supplement: Supplementary file 1 — Table 1. Simulation study results. The top half of the table shows the mean estimate of the average log‐odds ratio θ minus log(2), that is the bias of the estimate of θ; Monte Carlo standard errors are shown in parentheses. The bottom half of the table shows the mean estimate of τ2. The true value is θ=log(2) ≈0.693; results for θ=0 are shown in the main paper. Model 7* indicates that inferences for model 7 have been supplemented with results from the 'Peto approximation'. Table 2. Simulation study results. Actual coverage probability of 95% confidence intervals. The average model based standard errors, as a percentage of the corresponding empirical standard errors, are shown in parentheses. Model 7* indicates that inferences for model 7 have been supplemented with results from the 'Peto approximation' [file SIM-37-1059-s001.zip › measles.do-1.pdf]

```
// Analysis of measles data by 6 of Dan's 7 models
// IW 15mar2017

// Input data
version 13
clear
input str13 id  A     B    C     D
 "Anderson 1939"   4    43    6    43
    "Garly 2006"   1    43    6    32
    "Gibel 1942"   6    76    0   148
  "Hogarth 1939"   2   157    5   165
 "Karelitz 1951"   0    89    3    40
 "Karelitz 1954"   1   155   12    69
   "Prasad 1967"  13    64   27    53
end

// Reformat to 1 record per participant
rename A n11
rename B n10
rename C n01
rename D n00
reshape long n0 n1, i(id) j(event)
reshape long n, i(id event) j(treat)
drop if n==0
expand n
gen treat12=treat-0.5
encode id, gen(study)

// Fit the GLMMs

// In the GLMMs, intpoints() specifies the integration points for AGHQ
// Default is intpoints(7)
// In these models, choice of intpoints() affects the
//     3rd significant figure of the results,
//     but too large a choice makes convergence fail.
// The code below increases intpoints to 31 except where convergence fails

// Model 2
melogit event i.study treat || study: treat, nocons intpoints(31)

// Model 3
melogit event treat || study: treat, intpoints(15)

// Model 4
melogit event i.study treat || study: treat12, nocons intpoints(31)

// Model 5
melogit event treat || study: treat12, intpoints(31)

// Model 6
melogit event treat || study: treat12, cov(uns) intpoints(31)
```
