# Supplementary material for: A comparison of seven random‐effects models for meta‐analyses that estimate the summary odds ratio
Source: Stat Med. 2018 Jan 8;37(7):1059–85. doi: 10.1002/sim.7588 (PMC5841569; doi:10.1002/sim.7588)
Supplement: Supplementary file 1 — Table 1. Simulation study results. The top half of the table shows the mean estimate of the average log‐odds ratio θ minus log(2), that is the bias of the estimate of θ; Monte Carlo standard errors are shown in parentheses. The bottom half of the table shows the mean estimate of τ2. The true value is θ=log(2) ≈0.693; results for θ=0 are shown in the main paper. Model 7* indicates that inferences for model 7 have been supplemented with results from the 'Peto approximation'. Table 2. Simulation study results. Actual coverage probability of 95% confidence intervals. The average model based standard errors, as a percentage of the corresponding empirical standard errors, are shown in parentheses. Model 7* indicates that inferences for model 7 have been supplemented with results from the 'Peto approximation' [file SIM-37-1059-s001.zip › SAS output.pdf]

The SAS System

Obs	study	a	b	c	d
1	1	1	155	12	69
2	2	0	89	3	40
3	3	1	43	6	32
4	4	13	64	27	53
5	5	2	157	5	165
6	6	4	43	6	43
7	7	6	76	0	148

The SAS System

Obs	study	treat	n	event	control	treat12
1	1	0	81	12	1	-0.5
2	1	1	156	1	0	0.5
3	2	0	43	3	1	-0.5
4	2	1	89	0	0	0.5
5	3	0	38	6	1	-0.5
6	3	1	44	1	0	0.5
7	4	0	80	27	1	-0.5
8	4	1	77	13	0	0.5
9	5	0	170	5	1	-0.5
10	5	1	159	2	0	0.5
11	6	0	49	6	1	-0.5
12	6	1	47	4	0	0.5
13	7	0	148	0	1	-0.5
14	7	1	82	6	0	0.5

The SAS System

The GLIMMIX Procedure

Model Information	
Data Set	WORK.D2
Response Variable (Events)	event
Response Variable (Trials)	n
Response Distribution	Binomial
Link Function	Logit
Variance Function	Default
Variance Matrix Blocked By	study
Estimation Technique	Maximum Likelihood
Likelihood Approximation	Gauss-Hermite Quadrature
Degrees of Freedom Method	Containment

Class Level Information		
Class	Levels	Values
study	7	1 2 3 4 5 6 7

Number of Observations Read	14
Number of Observations Used	14
Number of Events	86
Number of Trials	1263

Dimensions	
G-side Cov. Parameters	1
Columns in X	9
Columns in Z per Subject	1
Subjects (Blocks in V)	7
Max Obs per Subject	2

Optimization Information	
Optimization Technique	Dual Quasi-Newton
Parameters in Optimization	9
Lower Boundaries	1
Upper Boundaries	0

**Fixed Effects**	Not Profiled
**Starting From**	GLM estimates
**Quadrature Points**	7

Iteration History					
**Iteration**	**Restarts**	**Evaluations**	**Objective Function**	**Change**	**Max Gradient**
**0**	**0**	4	70.979392293	.	5.895839
**1**	**0**	2	68.36477513	2.61461716	4.223485
**2**	**0**	3	68.130745504	0.23402963	6.859436
**3**	**0**	3	67.541459382	0.58928612	3.301563
**4**	**0**	4	65.716922986	1.82453640	4.139625
**5**	**0**	2	64.939420694	0.77750229	4.34333
**6**	**0**	2	64.43202308	0.50739761	2.058126
**7**	**0**	2	64.021000237	0.41102284	1.729085
**8**	**0**	3	63.803258306	0.21774193	0.745522
**9**	**0**	3	63.730666484	0.07259182	1.023047
**10**	**0**	2	63.641777234	0.08888925	0.373241
**11**	**0**	3	63.627812185	0.01396505	0.312859
**12**	**0**	4	63.538432189	0.08938000	0.071967
**13**	**0**	3	63.532592413	0.00583978	0.035598
**14**	**0**	3	63.532046864	0.00054555	0.021993
**15**	**0**	3	63.532008613	0.00003825	0.002299
**16**	**0**	3	63.532007254	0.00000136	0.000232
**17**	**0**	3	63.532007242	0.00000001	0.000033

Convergence criterion (GCONV=1E-8) satisfied.

Fit Statistics	
**-2 Log Likelihood**	63.53
**AIC (smaller is better)**	81.53
**AICC (smaller is better)**	126.53
**BIC (smaller is better)**	81.05
**CAIC (smaller is better)**	90.05
**HQIC (smaller is better)**	75.52

Fit Statistics for Conditional Distribution	

-2 log L(event | r. effects)	43.41
Pearson Chi-Square	2.10
Pearson Chi-Square / DF	0.15

Covariance Parameter Estimates

Cov Parm	Subject	Estimate	Standard Error
treat	study	3.2235	2.7079

Solutions for Fixed Effects

Effect	study	Estimate	Standard Error	DF	t Value	Pr > |t|
Intercept		-4.9507	1.0668	0	-4.64	.
study	1	3.1451	1.1312	0	2.78	.
study	2	2.1560	1.2797	0	1.68	.
study	3	3.2226	1.1691	0	2.76	.
study	4	4.2810	1.0892	0	3.93	.
study	5	1.4618	1.1485	0	1.27	.
study	6	3.0177	1.1302	0	2.67	.
study	7	0	.	.	.	.
treat		-1.2361	0.7818	6	-1.58	0.1649

Type III Tests of Fixed Effects

Effect	Num DF	Den DF	F Value	Pr > F
study	6	0	8.17	.
treat	1	6	2.50	0.1649

The SAS System

The GLIMMIX Procedure

Model Information	
Data Set	WORK.D2
Response Variable (Events)	event
Response Variable (Trials)	n
Response Distribution	Binomial
Link Function	Logit
Variance Function	Default
Variance Matrix Blocked By	study
Estimation Technique	Maximum Likelihood
Likelihood Approximation	Gauss-Hermite Quadrature
Degrees of Freedom Method	Containment

Class Level Information		
Class	Levels	Values
study	7	1 2 3 4 5 6 7

Number of Observations Read	14
Number of Observations Used	14
Number of Events	86
Number of Trials	1263

Dimensions	
G-side Cov. Parameters	2
Columns in X	2
Columns in Z per Subject	2
Subjects (Blocks in V)	7
Max Obs per Subject	2

Optimization Information	
Optimization Technique	Dual Quasi-Newton
Parameters in Optimization	4
Lower Boundaries	2
Upper Boundaries	0

**Fixed Effects**	Not Profiled
**Starting From**	GLM estimates
**Quadrature Points**	1

Iteration History

Iteration	Restarts	Evaluations	Objective Function	Change	Max Gradient
0	0	4	90.522602216	.	10.45531
1	0	2	87.269006361	3.25359585	3.122632
2	0	2	86.97763349	0.29137287	2.544907
3	0	4	86.001495032	0.97613846	0.74872
4	0	2	85.866272539	0.13522249	0.529515
5	0	2	85.802418642	0.06385390	0.480104
6	0	2	85.783378899	0.01903974	0.326114
7	0	2	85.756091719	0.02728718	0.041321
8	0	3	85.755485065	0.00060665	0.009445
9	0	3	85.755390289	0.00009478	0.001573
10	0	3	85.755389647	0.00000064	0.000377
11	0	3	85.755389625	0.00000002	8.041E-6

Convergence criterion (GCONV=1E-8) satisfied.

Fit Statistics

**-2 Log Likelihood**	85.76
**AIC (smaller is better)**	93.76
**AICC (smaller is better)**	98.20
**BIC (smaller is better)**	93.54
**CAIC (smaller is better)**	97.54
**HQIC (smaller is better)**	91.08

Fit Statistics for Conditional Distribution

**-2 log L(event | r. effects)**	46.83
**Pearson Chi-Square**	4.02
**Pearson Chi-Square / DF**	0.29

Covariance Parameter Estimates

Cov Parm	Subject	Estimate	Standard Error

**Intercept**	study	1.2816	0.8305
**treat**	study	2.3110	1.8719

Solutions for Fixed Effects

Effect	Estimate	Standard Error	DF	t Value	Pr > |t|
**Intercept**	-2.4290	0.4671	6	-5.20	0.0020
**treat**	-1.2414	0.6846	6	-1.81	0.1197

Type III Tests of Fixed Effects

Effect	Num DF	Den DF	F Value	Pr > F
**treat**	1	6	3.29	0.1197

The SAS System

The GLIMMIX Procedure

Model Information	
Data Set	WORK.D2
Response Variable (Events)	event
Response Variable (Trials)	n
Response Distribution	Binomial
Link Function	Logit
Variance Function	Default
Variance Matrix Blocked By	study
Estimation Technique	Maximum Likelihood
Likelihood Approximation	Gauss-Hermite Quadrature
Degrees of Freedom Method	Containment

Class Level Information		
Class	Levels	Values
study	7	1 2 3 4 5 6 7

Number of Observations Read	14
Number of Observations Used	14
Number of Events	86
Number of Trials	1263

Dimensions	
G-side Cov. Parameters	1
Columns in X	9
Columns in Z per Subject	1
Subjects (Blocks in V)	7
Max Obs per Subject	2

Optimization Information	
Optimization Technique	Dual Quasi-Newton
Parameters in Optimization	9
Lower Boundaries	1
Upper Boundaries	0

**Fixed Effects**	Not Profiled
**Starting From**	GLM estimates
**Quadrature Points**	7

Iteration History					
**Iteration**	**Restarts**	**Evaluations**	**Objective Function**	**Change**	**Max Gradient**
**0**	**0**	4	66.505680925	.	4.80791
**1**	**0**	3	65.929989278	0.57569165	9.356574
**2**	**0**	3	64.357306927	1.57268235	4.786486
**3**	**0**	3	63.484491888	0.87281504	2.276544
**4**	**0**	3	63.026030371	0.45846152	1.198231
**5**	**0**	3	62.945958216	0.08007216	1.121929
**6**	**0**	3	62.898428155	0.04753006	0.625674
**7**	**0**	2	62.817534119	0.08089404	0.320753
**8**	**0**	3	62.7959256	0.02160852	0.266417
**9**	**0**	2	62.762649461	0.03327614	0.180354
**10**	**0**	3	62.743543108	0.01910635	0.255284
**11**	**0**	3	62.736113836	0.00742927	0.072263
**12**	**0**	3	62.735797534	0.00031630	0.011383
**13**	**0**	3	62.735794403	0.00000313	0.000201
**14**	**0**	3	62.735794398	0.00000001	0.000029

Convergence criterion (GCONV=1E-8) satisfied.

Fit Statistics	
**-2 Log Likelihood**	62.74
**AIC (smaller is better)**	80.74
**AICC (smaller is better)**	125.74
**BIC (smaller is better)**	80.25
**CAIC (smaller is better)**	89.25
**HQIC (smaller is better)**	74.72

Fit Statistics for Conditional Distribution	
**-2 log L(event | r. effects)**	43.80
**Pearson Chi-Square**	2.47
**Pearson Chi-Square / DF**	0.18

Covariance Parameter Estimates			
**Cov Parm**	**Subject**	**Estimate**	**Standard Error**
**treat12**	study	2.6628	2.0730

Solutions for Fixed Effects						
**Effect**	**study**	**Estimate**	**Standard Error**	**DF**	**t Value**	**Pr > |t|**
**Intercept**		-3.3570	0.6819	0	-4.92	.
**study**	1	0.6579	0.6637	0	0.99	.
**study**	2	-0.2126	0.8882	0	-0.24	.
**study**	3	1.1975	0.7258	0	1.65	.
**study**	4	2.7261	0.5844	0	4.66	.
**study**	5	-0.1256	0.6977	0	-0.18	.
**study**	6	1.6498	0.6532	0	2.53	.
**study**	7	0	.	.	.	.
**treat**		-1.0241	0.7077	0	-1.45	.

Type III Tests of Fixed Effects				
**Effect**	**Num DF**	**Den DF**	**F Value**	**Pr > F**
**study**	6	0	10.93	.
**treat**	1	0	2.09	.

The SAS System

The GLIMMIX Procedure

Model Information	
Data Set	WORK.D2
Response Variable (Events)	event
Response Variable (Trials)	n
Response Distribution	Binomial
Link Function	Logit
Variance Function	Default
Variance Matrix Blocked By	study
Estimation Technique	Maximum Likelihood
Likelihood Approximation	Gauss-Hermite Quadrature
Degrees of Freedom Method	Containment

Class Level Information		
Class	Levels	Values
study	7	1 2 3 4 5 6 7

Number of Observations Read	14
Number of Observations Used	14
Number of Events	86
Number of Trials	1263

Dimensions	
G-side Cov. Parameters	2
Columns in X	2
Columns in Z per Subject	2
Subjects (Blocks in V)	7
Max Obs per Subject	2

Optimization Information	
Optimization Technique	Dual Quasi-Newton
Parameters in Optimization	4
Lower Boundaries	2
Upper Boundaries	0

**Fixed Effects**	Not Profiled
**Starting From**	GLM estimates
**Quadrature Points**	1

Iteration History					
**Iteration**	**Restarts**	**Evaluations**	**Objective Function**	**Change**	**Max Gradient**
**0**	**0**	4	85.049265313	.	2.905825
**1**	**0**	3	84.142959599	0.90630571	1.854907
**2**	**0**	2	83.625682823	0.51727678	3.053773
**3**	**0**	2	83.389074967	0.23660786	1.530378
**4**	**0**	2	83.201418742	0.18765623	0.259209
**5**	**0**	2	83.19536871	0.00605003	0.147549
**6**	**0**	3	83.191970129	0.00339858	0.13895
**7**	**0**	3	83.190513203	0.00145693	0.025912
**8**	**0**	3	83.190432794	0.00008041	0.001999
**9**	**0**	3	83.190431814	0.00000098	0.001431
**10**	**0**	3	83.190431631	0.00000018	6.431E-6

Convergence criterion (GCONV=1E-8) satisfied.

Fit Statistics	
**-2 Log Likelihood**	83.19
**AIC (smaller is better)**	91.19
**AICC (smaller is better)**	95.63
**BIC (smaller is better)**	90.97
**CAIC (smaller is better)**	94.97
**HQIC (smaller is better)**	88.52

Fit Statistics for Conditional Distribution	
**-2 log L(event | r. effects)**	45.01
**Pearson Chi-Square**	2.98
**Pearson Chi-Square / DF**	0.21

Covariance Parameter Estimates			
**Cov Parm**	**Subject**	**Estimate**	**Standard Error**
**Intercept**	study	0.9751	0.6028

**treat12**	study	2.7907	2.1526

Solutions for Fixed Effects					
**Effect**	**Estimate**	**Standard Error**	**DF**	**t Value**	**Pr > |t|**
**Intercept**	-2.4971	0.5300	6	-4.71	0.0033
**treat**	-1.0713	0.7170	0	-1.49	.

Type III Tests of Fixed Effects				
**Effect**	**Num DF**	**Den DF**	**F Value**	**Pr > F**
**treat**	1	0	2.23	.

The SAS System

The GLIMMIX Procedure

Model Information	
Data Set	WORK.D2
Response Variable (Events)	event
Response Variable (Trials)	n
Response Distribution	Binomial
Link Function	Logit
Variance Function	Default
Variance Matrix Blocked By	study
Estimation Technique	Maximum Likelihood
Likelihood Approximation	Gauss-Hermite Quadrature
Degrees of Freedom Method	Containment

Class Level Information		
Class	Levels	Values
study	7	1 2 3 4 5 6 7

Number of Observations Read	14
Number of Observations Used	14
Number of Events	86
Number of Trials	1263

Dimensions	
G-side Cov. Parameters	3
Columns in X	2
Columns in Z per Subject	2
Subjects (Blocks in V)	7
Max Obs per Subject	2

Optimization Information	
Optimization Technique	Dual Quasi-Newton
Parameters in Optimization	5
Lower Boundaries	2
Upper Boundaries	0

**Fixed Effects**	Not Profiled
**Starting From**	GLM estimates
**Quadrature Points**	1

Iteration History					
**Iteration**	**Restarts**	**Evaluations**	**Objective Function**	**Change**	**Max Gradient**
0	0	4	84.955775954	.	2.91077
1	0	3	84.132312645	0.82346331	1.686802
2	0	3	83.648084518	0.48422813	1.270122
3	0	3	83.501476539	0.14660798	1.479119
4	0	2	83.329566218	0.17191032	0.908377
5	0	3	83.267407593	0.06215863	0.551318
6	0	2	83.204444125	0.06296347	0.473104
7	0	3	83.185741696	0.01870243	0.121246
8	0	3	83.183311285	0.00243041	0.021624
9	0	3	83.183162579	0.00014871	0.003719
10	0	3	83.183159061	0.00000352	0.000429
11	0	3	83.183159023	0.00000004	0.000011

Convergence criterion (GCONV=1E-8) satisfied.

Fit Statistics	
**-2 Log Likelihood**	83.18
**AIC (smaller is better)**	93.18
**AICC (smaller is better)**	100.68
**BIC (smaller is better)**	92.91
**CAIC (smaller is better)**	97.91
**HQIC (smaller is better)**	89.84

Fit Statistics for Conditional Distribution	
**-2 log L(event | r. effects)**	45.01
**Pearson Chi-Square**	2.99
**Pearson Chi-Square / DF**	0.21

Covariance Parameter Estimates			
**Cov Parm**	**Subject**	**Estimate**	**Standard Error**

**UN(1,1)**	study	1.7417	1.2378
**UN(2,1)**	study	-1.4672	1.4075
**UN(2,2)**	study	2.7894	2.1569

Solutions for Fixed Effects

Effect	Estimate	Standard Error	DF	t Value	Pr > |t|
**Intercept**	-2.5023	0.5432	6	-4.61	0.0037
**treat**	-1.0560	0.7383	6	-1.43	0.2026

Type III Tests of Fixed Effects

Effect	Num DF	Den DF	F Value	Pr > F
**treat**	1	6	2.05	0.2026
