# Supplementary material for: A comparison of seven random‐effects models for meta‐analyses that estimate the summary odds ratio
Source: Stat Med. 2018 Jan 8;37(7):1059–85. doi: 10.1002/sim.7588 (PMC5841569; doi:10.1002/sim.7588)
Supplement: Supplementary file 1 — Table 1. Simulation study results. The top half of the table shows the mean estimate of the average log‐odds ratio θ minus log(2), that is the bias of the estimate of θ; Monte Carlo standard errors are shown in parentheses. The bottom half of the table shows the mean estimate of τ2. The true value is θ=log(2) ≈0.693; results for θ=0 are shown in the main paper. Model 7* indicates that inferences for model 7 have been supplemented with results from the 'Peto approximation'. Table 2. Simulation study results. Actual coverage probability of 95% confidence intervals. The average model based standard errors, as a percentage of the corresponding empirical standard errors, are shown in parentheses. Model 7* indicates that inferences for model 7 have been supplemented with results from the 'Peto approximation' [file SIM-37-1059-s001.zip › Simulation supplement1.pdf]

Research Article

Statistics in Medicine

Received XXXX

(www.interscience.wiley.com) DOI: 10.1002/sim.0000

Supplementary materials: Additional simulation study results

This supplementary document shows simulation study results for $\theta = \log(2) \approx 0.693$. The corresponding results for $\theta = 0$ are shown in the main paper. Note that in Table 1 we show the mean estimate of the average log-odds ratio θ minus log(2), that is the bias of the estimates of θ. This makes the results in Table 1 more directly comparable with those shown in the main paper. However we continue to show the mean estimates of τ^2.

Copyright © 0000 John Wiley & Sons, Ltd.

Table 1. Simulation study results. The top half of the table shows the mean estimate of the average log-odds ratio θ minus log(2), that is the bias of the estimate of θ; Monte Carlo standard errors are shown in parentheses. The bottom half of the table shows the mean estimate of τ^2. The true value is $\theta = \log(2) \approx 0.693$; results for $\theta = 0$ are shown in the main paper. Model 7* indicates that inferences for model 7 have been supplemented with results from the 'Peto approximation'.

Setting	Model 1 (D & L)	Model 1 (REML)	Model 2 (ML)	Model 3 (ML)	Model 4 (ML)	Model 5 (ML)	Model 6 (ML)	Model 7* (ML)
1 ($\mu = \log(2)$)	0.006 (0.083)	0.006 (0.083)	0.009 (0.084)	0.004 (0.084)	0.008 (0.084)	0.007 (0.083)	0.004 (0.084)	0.007 (0.083)
2 ($\mu = \log(2)$)	-0.004 (0.066)	-0.004 (0.066)	-0.002 (0.065)	-0.004 (0.066)	-0.001 (0.066)	-0.003 (0.065)	-0.002 (0.067)	-0.003 (0.066)
3 ($\mu = \log(2)$)	0.007 (0.152)	0.007 (0.151)	0.008 (0.153)	-0.001 (0.152)	0.009 (0.152)	0.010 (0.151)	-0.001 (0.152)	0.008 (0.152)
4 ($\mu = \log(2)$)	0.009 (0.156)	0.009 (0.158)	0.010 (0.156)	0.006 (0.156)	0.011 (0.157)	0.009 (0.157)	0.008 (0.159)	0.009 (0.157)
5 ($\mu = \log(2)$)	0.002 (0.116)	0.002 (0.115)	0.004 (0.115)	0.000 (0.115)	0.004 (0.116)	0.003 (0.115)	0.000 (0.118)	0.002 (0.115)
6 ($\mu = \log(2)$)	0.002 (0.059)	0.002 (0.059)	0.006 (0.060)	0.001 (0.059)	0.005 (0.059)	0.004 (0.059)	0.001 (0.059)	0.004 (0.059)
7 ($\mu = \log(2)$)	-0.005 (0.153)	-0.004 (0.154)	0.008 (0.157)	-0.005 (0.156)	0.009 (0.157)	0.002 (0.155)	-0.003 (0.157)	0.003 (0.156)
8 ($\mu = \log(2)$)	-0.003 (0.124)	-0.003 (0.124)	0.012 (0.126)	-0.001 (0.127)	0.011 (0.127)	0.009 (0.126)	0.003 (0.131)	0.011 (0.127)
9 ($\mu = \log(2)$)	-0.062 (0.213)	-0.060 (0.213)	0.013 (0.237)	-0.005 (0.240)	0.015 (0.240)	0.012 (0.239)	0.021 (0.257)	0.019 (0.247)
10 ($\mu = \log(2)$)	-0.002 (0.098)	-0.002 (0.098)	0.007 (0.098)	-0.001 (0.099)	0.006 (0.098)	0.004 (0.098)	0.001 (0.100)	0.005 (0.098)
11 ($\mu = \log(2)$)	0.001 (0.088)	0.001 (0.088)	0.006 (0.089)	0.000 (0.088)	0.006 (0.089)	0.004 (0.088)	0.001 (0.089)	0.005 (0.088)
12 ($\mu = \log(2)$)	0.003 (0.085)	0.003 (0.085)	0.009 (0.086)	0.018 (0.085)	0.008 (0.086)	0.017 (0.086)	0.019 (0.086)	0.007 (0.086)
13 ($\mu = \log(2)$)	0.000 (0.083)	0.000 (0.083)	0.002 (0.083)	-0.002 (0.083)	0.003 (0.083)	0.001 (0.083)	-0.001 (0.085)	0.002 (0.083)
14 ($\mu = \log(2)$)	-0.005 (0.087)	-0.005 (0.087)	-0.003 (0.088)	-0.007 (0.088)	-0.001 (0.088)	-0.003 (0.088)	-0.003 (0.088)	-0.002 (0.088)
15 ($\mu = \log(2)$)	-0.026 (0.431)	-0.019 (0.435)	-0.007 (0.446)	-0.007 (0.445)	-0.014 (0.440)	-0.020 (0.436)	-0.007 (0.446)	-0.013 (0.444)
1 ($\tau^2 = 0.024$)	0.027 (0.029)	0.026 (0.029)	0.005 (0.015)	0.020 (0.024)	0.020 (0.025)	0.021 (0.026)	0.023 (0.025)	0.021 (0.025)
2 ($\tau^2 = 0$)	0.007 (0.014)	0.007 (0.014)	0.000 (0.002)	0.005 (0.011)	0.004 (0.010)	0.005 (0.011)	0.008 (0.012)	0.005 (0.011)
3 ($\tau^2 = 0.168$)	0.163 (0.101)	0.166 (0.103)	0.113 (0.093)	0.147 (0.094)	0.144 (0.093)	0.148 (0.094)	0.148 (0.095)	0.144 (0.094)
4 ($\tau^2 = 0.024$)	0.037 (0.067)	0.038 (0.075)	0.007 (0.029)	0.018 (0.039)	0.017 (0.042)	0.017 (0.041)	0.030 (0.052)	0.017 (0.045)
5 ($\tau^2 = 0.024$)	0.029 (0.042)	0.030 (0.046)	0.006 (0.022)	0.020 (0.033)	0.018 (0.033)	0.019 (0.034)	0.026 (0.038)	0.019 (0.033)
6 ($\tau^2 = 0.024$)	0.026 (0.021)	0.025 (0.021)	0.003 (0.009)	0.022 (0.019)	0.022 (0.019)	0.023 (0.020)	0.023 (0.020)	0.023 (0.020)
7 ($\tau^2 = 0.024$)	0.050 (0.079)	0.047 (0.078)	0.003 (0.024)	0.035 (0.057)	0.037 (0.069)	0.039 (0.071)	0.063 (0.073)	0.039 (0.071)
8 ($\tau^2 = 0.024$)	0.037 (0.054)	0.036 (0.054)	0.002 (0.018)	0.028 (0.042)	0.024 (0.045)	0.032 (0.050)	0.043 (0.052)	0.030 (0.050)
9 ($\tau^2 = 0.024$)	0.029 (0.081)	0.029 (0.079)	0.001 (0.031)	0.039 (0.080)	0.043 (0.121)	0.066 (0.157)	0.107 (0.179)	0.078 (0.205)
10 ($\tau^2 = 0.024$)	0.029 (0.035)	0.029 (0.037)	0.001 (0.010)	0.023 (0.030)	0.014 (0.027)	0.024 (0.035)	0.028 (0.036)	0.022 (0.032)
11 ($\tau^2 = 0.024$)	0.029 (0.031)	0.029 (0.032)	0.002 (0.011)	0.022 (0.026)	0.018 (0.025)	0.023 (0.029)	0.026 (0.028)	0.022 (0.027)
12 ($\tau^2 = 0.024$)	0.029 (0.033)	0.028 (0.034)	0.003 (0.013)	0.021 (0.026)	0.018 (0.027)	0.023 (0.029)	0.026 (0.029)	0.022 (0.029)
13 ($\tau^2 = 0.024$)	0.029 (0.030)	0.029 (0.031)	0.006 (0.015)	0.022 (0.025)	0.022 (0.026)	0.023 (0.027)	0.025 (0.026)	0.022 (0.026)
14 ($\tau^2 = 0.024$)	0.027 (0.031)	0.027 (0.032)	0.004 (0.014)	0.018 (0.025)	0.020 (0.027)	0.021 (0.028)	0.024 (0.028)	0.021 (0.027)
15 ($\tau^2 = 2$)	1.388 (0.622)	1.944 (0.962)	1.808 (0.957)	1.830 (0.954)	1.767 (0.891)	1.726 (0.867)	1.828 (0.952)	1.811 (0.950)

Copyright © 0000 John Wiley & Sons, Ltd.

Prepared using simauth.cls

Table 2. Simulation study results. Actual coverage probability of 95% confidence intervals. The average model based standard errors, as a percentage of the corresponding empirical standard errors, are shown in parentheses. Model 7* indicates that inferences for model 7 have been supplemented with results from the 'Peto approximation'

Setting	Model 1 (D & L)	Model 1 (REML)	Model 2 (ML)	Model 3 (ML)	Model 4 (ML)	Model 5 (ML)	Model 6 (ML)	Model 7* (ML)
1	0.936 (99)	0.930 (99)	0.874 (80)	0.925 (94)	0.920 (94)	0.924 (95)	0.939 (96)	0.921 (95)
2	0.950 (105)	0.949 (105)	0.941 (98)	0.950 (103)	0.945 (102)	0.945 (103)	0.954 (105)	0.946 (103)
3	0.916 (94)	0.916 (95)	0.854 (80)	0.900 (90)	0.902 (89)	0.909 (91)	0.907 (90)	0.905 (89)
4	0.909 (101)	0.910 (100)	0.872 (82)	0.895 (90)	0.892 (89)	0.896 (90)	0.923 (98)	0.894 (90)
5	0.937 (102)	0.939 (102)	0.903 (84)	0.925 (96)	0.927 (95)	0.924 (95)	0.937 (100)	0.927 (96)
6	0.940 (99)	0.939 (99)	0.862 (78)	0.934 (96)	0.926 (96)	0.933 (97)	0.932 (97)	0.929 (97)
7	0.959 (105)	0.961 (104)	0.935 (92)	0.950 (100)	0.951 (99)	0.954 (100)	0.964 (106)	0.952 (100)
8	0.947 (102)	0.947 (102)	0.918 (87)	0.936 (97)	0.937 (96)	0.940 (98)	0.944 (101)	0.939 (97)
9	0.967 (118)	0.968 (118)	0.956 (100)	0.964 (104)	0.961 (104)	0.962 (105)	0.970 (108)	0.962 (105)
10	0.928 (99)	0.924 (99)	0.880 (82)	0.916 (96)	0.903 (90)	0.916 (96)	0.929 (98)	0.912 (95)
11	0.949 (102)	0.947 (102)	0.895 (81)	0.946 (98)	0.929 (95)	0.941 (99)	0.947 (99)	0.942 (98)
12	0.944 (105)	0.940 (104)	0.898 (85)	0.932 (100)	0.931 (97)	0.934 (101)	0.946 (103)	0.936 (100)
13	0.933 (101)	0.935 (101)	0.880 (82)	0.925 (97)	0.921 (96)	0.924 (97)	0.929 (97)	0.929 (97)
14	0.929 (100)	0.930 (100)	0.889 (83)	0.924 (94)	0.920 (96)	0.924 (96)	0.935 (99)	0.921 (96)
15	0.890 (86)	0.918 (100)	0.898 (94)	0.900 (95)	0.900 (94)	0.899 (94)	0.900 (95)	0.900 (95)

Copyright © 0000 John Wiley & Sons, Ltd.
