# Supplementary material for: A comparison of seven random‐effects models for meta‐analyses that estimate the summary odds ratio
Source: Stat Med. 2018 Jan 8;37(7):1059–85. doi: 10.1002/sim.7588 (PMC5841569; doi:10.1002/sim.7588)
Supplement: Supplementary file 1 — Table 1. Simulation study results. The top half of the table shows the mean estimate of the average log‐odds ratio θ minus log(2), that is the bias of the estimate of θ; Monte Carlo standard errors are shown in parentheses. The bottom half of the table shows the mean estimate of τ2. The true value is θ=log(2) ≈0.693; results for θ=0 are shown in the main paper. Model 7* indicates that inferences for model 7 have been supplemented with results from the 'Peto approximation'. Table 2. Simulation study results. Actual coverage probability of 95% confidence intervals. The average model based standard errors, as a percentage of the corresponding empirical standard errors, are shown in parentheses. Model 7* indicates that inferences for model 7 have been supplemented with results from the 'Peto approximation' [file SIM-37-1059-s001.zip › Supplementary1_ml_aug8.pdf]

Supplementary materials: R codes to produce the results in Section 5.

Cut and paste the codes below to produce the results. Three required R packages are loaded in the first three commands. Version 1.9-9 of metafor was used. The results can be sensitive to the version of R packages that are used; Version 1.9-9 was the latest version when these supplementary materials were produced. A few additional results to those shown in the paper are also produced by these codes.

```
library(metafor)
library(BiasedUrn)
library(lme4)

##################### FUNCTIONS ###################

################# golong: Converts data into long format
golong <- function(dat)
  {
  n <- c(dat$A+dat$B, dat$C+dat$D)
  event <- c(dat$A, dat$C)
  study <- c(1:nrow(dat), 1:nrow(dat))
  obs <- 1:length(n)
  treat <- c(rep(1, length(n)/2), rep(0, length(n)/2))
  control <- 1-treat
  treat12 <- treat - 0.5
  dat_long <- data.frame(n, event, study, obs, treat, control,
treat12)
  dat_long
  }

################## Error function #####################

errorfun <- function(cond) {
  message("Error occurs in the code. Here's the original error
message:")
  message(cond)
  # Choose a return value in case of error
  return(list(b=NA, se=NA, tau2=NA))
}

############### fit_mods: Identical to fit_mods used in simulation
############### study, but with data inputted in a different manner

fit_mods <- function(the_dat, the_dat_long, the_dat_PETO, NAGQ=7)
{
# Model 1 (DL):
  DL <- rma.uni(yi=yi, vi=vi, method="DL",data=the_dat)

# Model 1 (REML):
  REML <- rma.uni(yi=yi, vi=vi, method="REML",data=the_dat)

# Model 2:
```

```
  HS <- glmer(cbind(event,n-event) ~ factor(treat) + factor(study) +
(treat-1|study),data=the_dat_long, family=binomial(link="logit"),
nAGQ=NAGQ)

# Model 3
  HS.random <- glmer(cbind(event,n-event) ~ factor(treat) + (1|study)
+ (treat-1|study),data=the_dat_long, family=binomial(link="logit"),
nAGQ=1)

# Model 4:
  UMFS <- rma.glmm(measure="OR", ai=A, bi=B, ci=C, di=D, data=the_dat,
model="UM.FS", nAGQ=NAGQ, drop00 = F)

# Model 5:
  UMRS <- rma.glmm(measure="OR", ai=A, bi=B, ci=C, di=D, data=the_dat,
model="UM.RS", nAGQ=1, drop00 = F)

# Model 6
  VanH<-glmer(cbind(event,n-event) ~ treat + (control + treat-
1|study),  data=the_dat_long, family=binomial(link="logit"), nAGQ=1)

# Model 7:
  CMEL <- tryCatch({rma.glmm(measure="OR", ai=A, bi=B, ci=C, di=D,
dat=the_dat, model="CM.EL", nAGQ=NAGQ, drop00 = F)}, error=errorfun)

# Model 7 (with changes to default):

  CMELhess <- tryCatch({rma.glmm(measure="OR", ai=A, bi=B, ci=C, di=D,
dat=the_dat, model="CM.EL", drop00 = F,
control=list(optCtrl=list(maxit=20000, reltol=0.0001),
optmethod="Nelder-Mead", hessianCtrl=list(r=16)), nAGQ=NAGQ)},
error=errorfun)

# Model 7 (approximate likelihood)
  CMAL <- rma.glmm(measure="OR", ai=A, bi=B, ci=C, di=D, data=the_dat,
model="CM.AL", nAGQ=NAGQ, drop00 = F)

# Peto methods (not considered):
  PETO<- rma.uni(yi=yi, vi=vi, method="ML",data=the_dat_PETO)

  PETO_DL<- rma.uni(yi=yi, vi=vi, method="DL",data=the_dat_PETO)


# Estimates, standard errors, tau2 estimates:

# Model 1 (DL):
estDL <- as.numeric(DL$b)
seDL <- as.numeric(DL$se)
tau2DL <- as.numeric(DL$tau2)

# Model 1 (REML):
estREML <- as.numeric(REML$b)
```

```
seREML <- as.numeric(REML$se)
tau2REML<- as.numeric(REML$tau2)

# Model 2:
estHS <- as.numeric(summary(HS)$coeff[2,1])
seHS <- as.numeric(summary(HS)$coeff[2,2])
tau2HS <- as.numeric(summary(HS)$varcor)

# Model 3:
estHS.random <- as.numeric(summary(HS.random)$coeff[2,1])
seHS.random <- as.numeric(summary(HS.random)$coeff[2,2])
tau2HS.random <- as.numeric(summary(HS.random)$varcor)[2]

# Model 4:
estUMFS <- as.numeric(UMFS$b)
seUMFS <- as.numeric(UMFS$se)
tau2UMFS <- as.numeric(UMFS$tau2)

# Model 5:
estUMRS <- as.numeric(UMRS$b)
seUMRS <- as.numeric(UMRS$se)
tau2UMRS <- as.numeric(UMRS$tau2)

# Model 6:
estVH <- as.numeric(summary(VanH)$coeff[2,1])
seVH <- as.numeric(summary(VanH)$coeff[2,2])
Bet_mat <- matrix(as.numeric(summary(VanH)$varcor$study), ncol=2)
tau2VH <- Bet_mat[1,1] + Bet_mat[2,2] - 2*Bet_mat[1,2]

# Model 7:
estCMEL <- as.numeric(CMEL$b)
seCMEL <- as.numeric(CMEL$se)
tau2CMEL <- as.numeric(CMEL$tau2)

# Model 7 (with changes to default):
estCMELhess <- as.numeric(CMELhess$b)
seCMELhess <- as.numeric(CMELhess$se)
tau2CMELhess <- as.numeric(CMELhess$tau2)

# Model 7 (approximate likelihood):
estCMAL <- as.numeric(CMAL$b)
seCMAL <- as.numeric(CMAL$se)
tau2CMAL <- as.numeric(CMAL$tau2)

# Peto methods (not considered):
estPETO <- as.numeric(PETO$b)
sePETO <- as.numeric(PETO$se)
tau2PETO <-as.numeric(PETO$tau2)

estPETO_DL <- as.numeric(PETO_DL$b)
sePETO_DL <- as.numeric(PETO_DL$se)
tau2PETO_DL <- as.numeric(PETO_DL$tau2)
```

```
  estimates <- c(estDL, estREML, estHS, estHS.random, estUMFS, 
estUMRS, estVH, estCMEL, estCMELhess, estCMAL, estPETO, estPETO_DL)

  ses <- c(seDL, seREML, seHS, seHS.random,  seUMFS, seUMRS,seVH, 
seCMEL,  seCMELhess, seCMAL, sePETO, sePETO_DL)

  tau2s <- c(tau2DL, tau2REML, tau2HS, tau2HS.random,  tau2UMFS, 
tau2UMRS, tau2VH, tau2CMEL, tau2CMELhess, tau2CMAL,  tau2PETO, 
tau2PETO_DL)

  return(list(estimates=estimates, ses=ses, tau2s=tau2s))
}


######################### END OF FUNCTIONS ########################





###################### LOAD DATA ###########################

# Outcome 1:
karelitz1954 <- c(1, 156-1, 12, 81-12)
karelitz1951 <- c(0, 89-0, 3, 43-3)
garly2006 <- c(1, 44-1, 6, 38-6)
prasad1967 <- c(13, 77-13, 27, 80-27)
hogarth1939 <- c(2, 159-2, 5, 170-5)
anderson1939 <- c(4, 47-4, 6, 49-6)
gibel1942 <- c(6, 82-6, 0, 148)
ns1 <- rbind(karelitz1954, karelitz1951, garly2006, prasad1967, 
hogarth1939, anderson1939, gibel1942)


anderson1939 <- c(5, 50-5, 12, 50-12)
garly2006 <- c(2, 44-2, 5, 37-5)
hogarth1939 <- c(8, 159-8, 7, 170-7)
karelitz1954 <- c(0, 175-0, 1, 81-1)
ns2 <- rbind(anderson1939, garly2006, hogarth1939, karelitz1954)


anderson1939 <- c(3, 60-3, 7, 59-7)
garly2006 <- c(1, 44-1, 2, 37-2)
gibel1942 <- c(0, 195-0, 0, 180-0)
hogarth1939 <- c(5, 159-5, 12, 170-12)
karelitz1951 <- c(1, 86-1, 0, 43-5)
ns4 <- rbind(anderson1939, garly2006, gibel1942, hogarth1939, 
karelitz1951)


# Outcome 4 with double-zero study removed:
```

```
anderson1939 <- c(3, 60-3, 7, 59-7)
garly2006 <- c(1, 44-1, 2, 37-2)
hogarth1939 <- c(5, 159-5, 12, 170-12)
karelitz1951 <- c(1, 86-1, 0, 43-5)
ns4b <- rbind(anderson1939, garly2006, hogarth1939, karelitz1951)


ns <- list(ns1, ns2, ns4, ns4b)

dat <- list()
datlong <- list()
dat_PETO <- list()

########################## PUT THE DATA INTO THE CORRECT FORMAT
for(i in 1:length(ns))
  {
  colnames(ns[[i]]) <- c("A", "B", "C", "D")
  dat[[i]] <- escalc(measure="OR", ai=A, bi=B, ci=C, di=D,
data=ns[[i]], digits=8)
  datlong[[i]] <- golong(dat[[i]])
  dat_PETO[[i]] <- escalc(measure="PETO", ai=A, bi=B, ci=C, di=D,
data=ns[[i]], digits=8)
  }


################################# FIT THE MODELS

output <- list()

for(i in 1:length(dat))
  {
  output[[i]] <- fit_mods(dat[[i]], datlong[[i]], dat_PETO[[i]],
NAGQ=7)
  }


############################### ORGANISE THE RESULTS INTO TABLES

est <- matrix(NA, nrow=12, ncol=4)#,
colnames(est) <- c("ma1", "ma2", "ma4", "ma4b")
rownames(est) <- c("DL", "REML", "HS", "HSrand", "UMFS", "UMRS",
"VH","CMEL", "CMELhess", "CMAL",   "PETO", "PETO_DL")

se <- est
tau2 <- est

for(i in 1:length(dat))
  {
  est[,i] <- output[[i]]$estimates
  se[,i] <- output[[i]]$ses
  tau2[,i] <- output[[i]]$tau2s
  }
```

```
# Results:
round(est, 3)
round(se, 3)
round(tau2, 3)
# ^^^ Note:
# - small standard error for model 3 (HS random), outcome 1;
# - No result for model 7 (CM.EL), outcomes 4 and 4b.


# Change from defaults for model 3, outcome 1:

HSrand.1.1 <- glmer(cbind(event,n-event) ~ factor(treat) + (1|study) +
(treat-1|study),data=datlong[[1]], family=binomial(link="logit"),
 control=glmerControl(optCtrl=list(maxfun=10e9)))

summary(HSrand.1.1)

# Insert this into table:
est["HSrand", "ma1"] <- summary(HSrand.1.1)$coeff[2,1]
se["HSrand", "ma1"] <- summary(HSrand.1.1)$coeff[2,2]
tau2["HSrand", "ma1"] <- as.numeric(summary(HSrand.1.1)$varcor)[2]

##### Use non-default options, i.e. CMELhess results, for model 7,
##### outcomes 4 and 4b:

est["CMEL", "ma4"] <- est["CMELhess", "ma4"]
se["CMEL", "ma4"] <- se["CMELhess", "ma4"]
tau2["CMEL", "ma4"] <- tau2["CMELhess", "ma4"]

est["CMEL", "ma4b"] <- est["CMELhess", "ma4b"]
se["CMEL", "ma4b"] <- se["CMELhess", "ma4b"]
tau2["CMEL", "ma4b"] <- tau2["CMELhess", "ma4b"]




# Updated results incorporating results directly above, and including
models 1-7 only:
round(est[c("DL", "REML", "HS", "HSrand", "UMFS", "UMRS", "VH",
"CMEL"), ], 3)
round(se[c("DL", "REML", "HS", "HSrand", "UMFS", "UMRS", "VH",
"CMEL"), ], 3)
round(tau2[c("DL", "REML", "HS", "HSrand", "UMFS", "UMRS", "VH",
"CMEL"), ], 3)
```
