# Supplementary material for: A comparison of seven random‐effects models for meta‐analyses that estimate the summary odds ratio
Source: Stat Med. 2018 Jan 8;37(7):1059–85. doi: 10.1002/sim.7588 (PMC5841569; doi:10.1002/sim.7588)
Supplement: Supplementary file 1 — Table 1. Simulation study results. The top half of the table shows the mean estimate of the average log‐odds ratio θ minus log(2), that is the bias of the estimate of θ; Monte Carlo standard errors are shown in parentheses. The bottom half of the table shows the mean estimate of τ2. The true value is θ=log(2) ≈0.693; results for θ=0 are shown in the main paper. Model 7* indicates that inferences for model 7 have been supplemented with results from the 'Peto approximation'. Table 2. Simulation study results. Actual coverage probability of 95% confidence intervals. The average model based standard errors, as a percentage of the corresponding empirical standard errors, are shown in parentheses. Model 7* indicates that inferences for model 7 have been supplemented with results from the 'Peto approximation' [file SIM-37-1059-s001.zip › Supplementary material SAS code.pdf]

```
/* Supplementary materials: SAS code to produce the results in Section 5 for outcome 1 */

/* We give the SAS code for models 2-6. Model 1 (DerSimonian and Laird) is not available
in SAS, though there are user supplied macro's. Model 7 can be fitted in SAS Proc NLMIXED.
For the NLMIXED code we refer to Stijnen et al (Stat Med 2010) */

/* read in the data */
data d1;input study a b c d;
datalines;
1 1 155 12 69
2 0 89 3 40
3 1 43 6 32
4 13 64 27 53
5 2 157 5 165
6 4 43 6 43
7 6 76 0 148
;

/* make a long format data set */
data d2;set d1;
treat=0;n=c+d;event=c;control=1;treat12=treat-0.5;output;
treat=1;n=a+b;event=a;control=0;treat12=treat-0.5;output;
keep study treat event n control treat12;
proc print;run;

* model two *;
proc glimmix data=d2 method=quad(qpoints=7);
class study;
model event/n = study treat / solution;
random treat / subject=study;
run;

* model three *;
proc glimmix data=d2 method=quad(qpoints=1);
class study;
model event/n = treat / solution;
random intercept treat / subject=study type=vc;
run;

* model four *;
proc glimmix data=d2 method=quad(qpoints=7);
class study;
model event/n = study treat / solution;
random treat12 / subject=study;
run;

* model five *;
proc glimmix data=d2 method=quad(qpoints=1);
class study;
model event/n = treat / solution;
random intercept treat12 / subject=study type=vc;
run;
```

```
* model six *;
proc glimmix data=d2 method=quad(qpoints=1);
class study;
model event/n = treat / solution;
random intercept treat / subject=study type=un;
run;
```
