# Supplementary material for: A comparison of seven random‐effects models for meta‐analyses that estimate the summary odds ratio
Source: Stat Med. 2018 Jan 8;37(7):1059–85. doi: 10.1002/sim.7588 (PMC5841569; doi:10.1002/sim.7588)
Supplement: Supplementary file 1 — Table 1. Simulation study results. The top half of the table shows the mean estimate of the average log‐odds ratio θ minus log(2), that is the bias of the estimate of θ; Monte Carlo standard errors are shown in parentheses. The bottom half of the table shows the mean estimate of τ2. The true value is θ=log(2) ≈0.693; results for θ=0 are shown in the main paper. Model 7* indicates that inferences for model 7 have been supplemented with results from the 'Peto approximation'. Table 2. Simulation study results. Actual coverage probability of 95% confidence intervals. The average model based standard errors, as a percentage of the corresponding empirical standard errors, are shown in parentheses. Model 7* indicates that inferences for model 7 have been supplemented with results from the 'Peto approximation' [file SIM-37-1059-s001.zip › Two additional real examples.pdf]

Two additional real examples

In these web supplementary materials we consider two additional real examples.

Example one

The first example is analysis 1.4 from the Cochrane Review “Exercise for osteoarthritis of the knee” by Fransen et al (DOI: 10.1002/14651858.CD004376.pub3). Here we compare exercise to control, where the outcome is study withdrawal. A negative estimate of theta favours the treatment. 45 studies contribute to the analysis, so that we can anticipate that models will be better identified than those in the main paper. However both the estimate of the log-odds ratio and the between-study variance are quite sensitive to the model used (see the table of results) and we conclude that sensitivity analyses retain their usefulness even in examples like this one with many studies.

Example two

The second example is analysis 1.1 from the Cochrane Review “Immunosuppressive treatment for idiopathic membranous nephropathy in adults with nephrotic syndromeby” by Chen et al (DOI: 10.1002/14651858.CD004293.pub3.). Here we compare Immunosuppressive treatments versus placebo/no treatment/nonimmunosuppressive treatments, and the outcome is death or ESKD (dialysis/transplantation) (ITT analysis). As in the previous example, a negative estimate of theta favours the treatment. 17 studies contribute to the analysis but 5 are double zero studies. We show results in our table for this example where we both include and exclude “double-zero” studies; the latter set of results are shown in the table as Example 2 (omit 00).

We obtain fairly robust evidence that the treatment is effective. We conclude that models 2, 4 and 7 are not sensitive to the decision of whether or not to include the “double-zero” studies (the slightly larger standard error from model 4 when excluding these studies can be attributed to numerical error). Models 3, 5 and 6 are sensitive to this decision. However they are not very sensitive to this decision, despite the fact that there are 5/17 studies where no events occur. The factors determining when models 3, 5 and 6 will be sensitive to this decision are likely to be complicated and are worthy of further study. As explained in the main paper, some artificially small standard errors had to be corrected for this example (when double-zero studies were excluded) by increasing the number of function evaluations allowed.

Table of results

Theta	Example 1	Example 2	Example 2 (omit 00)
Model 1 (D & L)	-0.072 (0.109)	-0.644 (0.235)	-0.656 (0.312)
Model 1 (REML)	-0.069 (0.111)	-0.603 (0.288)	-0.654 (0.316)
Model 2	-0.070 (0.088)	-0.767 (0.230)	-0.767 (0.230)
Model 3	-0.080 (0.101)	-0.777 (0.286)	-0.767 (0.267)*
Model 4	-0.031 (0.116)	-0.716 (0.300)	-0.716 (0.302)
Model 5	-0.053 (0.111)	-0.679 (0.304)	-0.696 (0.301)*
Model 6	0.009 (0.115)	-0.498 (0.400)	-0.614 (0.313)
Model 7	-0.027 (0.115)	-0.711 (0.313)	-0.711 (0.313)
Tau-squared			
Model 1 (D & L)	0.094	0	0.299
Model 1 (REML)	0.106	0.243	0.320
Model 2	0	0	0
Model 3	0.072	0.232	0.151*
Model 4	0.155	0.285	0.285
Model 5	0.138	0.326	0.306*
Model 6	0.124	0.317	0.262
Model 7	0.165	0.344	0.344

Data set for example 1

A	B	C	D
3	11	4	10
1	22	1	22
2	15	2	15
13	60	2	65
6	39	7	37
6	19	6	19
4	18	6	13
8	20	7	6
6	24	13	17
9	33	5	36
11	61	14	56
26	120	23	126
3	32	3	32
6	14	3	25
3	40	2	41
4	52	0	41
0	17	2	6
16	93	19	89
8	91	0	33
5	30	3	32
12	68	10	60

49	229	27	113
3	65	4	30
5	66	4	31
21	61	11	65
20	114	34	91
0	16	2	16
5	47	6	45
1	28	2	13
4	49	6	48
2	70	3	33
5	20	3	24
8	49	7	49
5	108	6	72
10	59	3	65
1	11	1	12
6	13	3	15
3	47	1	24
1	10	1	11
6	4	10	21
2	15	4	13
2	28	3	28
6	93	4	98
2	24	2	24
24	64	41	53

Dataset for example 2

A	B	C	D
9	43	10	41
6	58	5	51
1	33	7	31
0	11	0	11
1	12	0	13
0	27	3	24
2	13	1	10
3	39	12	27
6	45	19	34
0	4	0	5
2	7	4	4
3	7	1	10
3	19	1	10
0	5	0	5
0	25	1	22
0	19	0	17
0	5	0	4
